# Supplementary material for: Evaluating the effect of short-course rifapentine-based regimens with or without enhanced behaviour-targeted treatment support on adherence and completion of treatment for latent tuberculosis infection among adults in the UK (RID-TB: Treat): protocol for an open-label, multicentre, randomised controlled trial
Source: BMJ Open. 2022 Sep 6;12(9):e057717. doi: 10.1136/bmjopen-2021-057717 (PMC9454004; doi:10.1136/bmjopen-2021-057717)
Supplement: Supplementary data [file bmjopen-2021-057717supp001.pdf]

Summary Participant Information Sheet for the RID-TB:Treat Clinical Trial

We are inviting you to take part in a research study called RID-TB:Treat.

This study aims to help people complete a course of medicines for treatment of latent tuberculosis infection or LTBI. If you have LTBI, it means you have been infected with the bacteria that cause tuberculosis (TB), but you are not ill and you do not have any symptoms. Treatment for LTBI can prevent TB.

This study is part of RID-TB, a 5–year work programme that includes several linked studies on LTBI. At this stage you are only being asked to take part in RID-TB:Treat.

This page gives you an overview of the study. Please take time to read the whole leaflet carefully so you can decide if you would like to take part. You can share this information sheet and discuss the research study with friends and relatives if you wish.

The study in brief:

This study aims to find out:

- Whether taking different LTBI medicines for a shorter amount of time (one month) than normal (three months), or once a week rather than once a day, makes it easier for people to take all of their medication.
- Whether using a pill box and reminders to take medicines as well as support materials helps people to remember to take their medicine and not miss any doses (we call this adherence support).

In addition, we would also like to find out through the following optional substudies:

- Behavioural substudy: Your thoughts, feelings, and experiences around treatment of LTBI and adherence support
- Health economics substudy: How much treatment of LTBI costs and how the treatment affects your life. This will allow us to see if new LTBI treatment and/or additional adherence support offers value for money for the NHS.

The behavioural and economics substudies are optional, include only people who agree, and will involve completeing simple tick-box questions and interviews for selected individuals.

Key points you need to know:

This study investigates the use of three medicines used in two different combinations. Each combination is given with or without ‘adherence support’, which includes educational messages and reminders to take medicines.

There are six different groups within the study. You could be allocated to any of these groups:

GROUP 1 - Daily isoniazid + rifampicin for three months (3HR), routine adherence support (standard-of-care)

GROUP 2 - Daily isoniazid + rifampicin for three months (3HR), additional adherence support

GROUP 3 -Weekly isoniazid + rifapentine for three months (3HP), routine adherence support

GROUP 4 - Weekly isoniazid + rifapentine for three months (3HP), additional adherence support

GROUP 5 - Daily isoniazid + rifapentine for one month (1HP), routine adherence support

GROUP 6 - Daily isoniazid + rifapentine for one month (1HP), additional adherence support.

We are studying two new types of LTBI treatment: one month of daily medicines (1HP)

Rangaka MX, *et al. BMJ Open* 2022; 12:e057717. doi: 10.1136/bmjopen-2021-057717

and three months of medicines which need to be taken once a week (3HP) . These two new types of LTBI treatment will be compared to three months of daily treatment (3HR) usually used in the UK (standard-of-care). We think the medicines should be equally effective and safe. We want to find out whether either of these ways of taking the medicines help you complete a course without missing any doses.

All LTBI medicines, including those used in this trial, can have unwanted side effects. They are usually minor and reversible, if they occur at all. The most common are allergic reactions, flu-like symptoms, headache, skin reactions, diarrhoea, liver problems, nausea, vomiting and a decrease in white blood cell and red blood cell count.

We are also testing whether reminders to take pills and adherence support materials will help you to follow your treatment schedule. You will get a special pill box which will record each time it is opened.

This study will *not* require you to visit the hospital more times than if you were being treated in the usual way for latent TB.

What happens if I am interested in taking part?

If you agree to take part in the study after reading all the information, we will check your medical records. This is to see whether you meet the study entry criteria and check it is safe for you to do take part. We will ask you to sign a consent form and will give you copies of both this information sheet and the consent form. We will also write to your GP to let them know that you have agreed to take part in this research, this is optional and you can opt for your GP not to be informed.

If you do not wish to take part in the study, or if you do not meet the study entry criteria, you are likely to receive the standard-of-care treatment, which is daily antibiotics for three months and usual support to help you remember to take your medicines.

You are free to decide whether to take part in this research study or not. If you choose not to take part, this will not affect the care you receive.

If you do agree, you can stop taking part in the study at any time, without giving a reason.

Please ask your doctor or nurse if there is anything that unclear or if you would like more information.

If you have any questions about this study, please talk to your doctor or nurse:

Name of doctor or nurse:

Hospital Department:

Hospital:

Address:

Tel: 01234 XXX XXX

Email: (if applicable)

1. Why are we doing this study?

This study aims to help people complete prescribed medicines for treatment of latent TB infection (LTBI) and ensure its treatment for LTBI for latent TB works best when taken as prescribed. This study aims to find the best way to support people to take LTBI treatment

What are we trying to find out?

This study aims to find out whether taking different LTBI medicines for a shorter amount of time than normal, or once a week rather than daily, makes it easier for people to take all of their medication and not miss any doses.

We also want to know whether a support package that we have developed which includes a video animation, a pill box and text

message reminders can help people to take their medicines.

2. What is latent TB?

If you have latent TB infection (LTBI) it means you have been infected with the bacteria that causes tuberculosis (TB), but you are not ill and you do not have any symptoms. If you then become ill with "active" TB disease you could pass TB on to other people. TB bacteria are spread through the air, mainly by coughing. If you want to know more about latent TB talk to the doctor or nurse who is treating you.

How is latent TB usually treated?

Active TB can be cured with a combination of different antibiotics, which need to be taken for many months (at least 6 months). LTBI can be diagnosed and treated to help prevent TB disease from developing. The treatment for latent TB in England is usually 3 months long and fewer drugs are given compared with active disease.

3. Why am I being asked to take part?

You are being asked to take part in the RID-TB:Treat study because you have latent TB and treatment is recommended

Your doctor will perform an assessment and tests that are routinely required before treatment of LTBI for your safety. We will check if you can take partn the study using these results.

To take part in RID-TB:Treat :

- You will be diagnosed with LTBI
- You will be between 16 and 65 years of age
- You will not have signs of active TB (this includes
- You will not have a known allergy to the medicines in the study
- You will not have any liver problems that might mean you can't take the medicines safely (a blood test will be done to check this)
- You will not be pregnant or breastfeeding, or plan on becoming pregnant during the study
- Females who are able to become pregnant (of child-bearing potential) will agree to using contraception whilst on the medications (specifically, an implant or male partners using condoms. Oral contraceptives may be less effective whilst on treatment)
- Male whose female partners are able to become pregnant will agree to using contraception whilst on the medications.

4. What do I need to know about the medicines in this study?

The new LTBI medicine we want to find out more about in this study is called rifapentine, which is given in combination with another medicine called isoniazid. You will only receive rifapentine if you are in Group 3, 4, 5 or 6. There are different ways of taking this tablet: once a day for a month (1HP), or once a week for three months (3HP). It is the same dose each intake. You swallow these tablets within one hour after eating food.

An often-used treatment for LTBI is a medicine combining both rifampicin and isoniazid in a single tablet. You will receive this medicine if you are in Group 1 or 2. This medication is taken once a day, for three months (3HR). You swallow this tablet on an empty stomach (at least 30 minutes before food or 2 hours after food.)

We will also investigate whether additional adherence support helps people to take their medicine. This includes a reminder via SMS message or sound alarm using the pill box and adherence support materials such as video

animation. This support will be given in addition to usual support by clinicians.

What are the possible side-effects?

All medicines can have unwanted side-effects, including those normally used for LTBI treatment outside of this study. The most common side-effects of rifapentine, rifampicin and isoniazid are: allergic reactions and flu-like symptoms, headache, skin reactions, diarrhoea, liver problems, nausea, vomiting, and decrease in white blood cell and red blood cell count.

A common side effect rifapentine and rifampicin may cause a temporary red-orange staining of body tissues or fluids. This would include skin, teeth, tongue, urine, faeces, saliva, sputum, tears, sweat, and breast milk. Contact lenses or dentures may become permanently stained.

If you become concerned about any side-effects, please tell your doctor or nurse as soon as possible.

5. What are the possible benefits of taking part in this study?

We hope that you will directly benefit from the medicines used in this study and from the tools used to help you with treatment adherence, but we cannot guarantee this. However, the information we will collect from this study will help us to improve future treatments for people like you diagnosed with LTBI in the future.

6. What will I need to do if I take part? Can I definitely take part?

Not everyone may be able to take part in this study. We will first check whether you are suitable for the trial by taking a medical history, checking your symptoms and assessing your physical health. We will also check results of tests which are routinely performed before treatment of latent TB.

If you agree to be part of the trial, you will also be agreeing to:

- Having some tests to check you can take part in the trial. These include, a blood test and a pregnancy test, if you are a woman who is able to become pregnant.
- Receiving any of the six groups of LTBI treatments. To make this a fair test, you cannot choose and must be happy to accept any of the groups of treatment.
- For us to collect your information whilst you are on the study

What if the tests show I can take part?

If these tests show you can take part and you agree to join the RID-TB-Treat study, we will ask you to sign a consent form. There will be six different groups in this study.

Which group will I be in?

It is important that the groups receiving each treatment are as similar as possible at the start of the study. To ensure that this happens, a process called randomisation is used to allocate people to each group. This means, a computer will randomly select which group you are in, like “the toss of a coin”. Your doctor will offer you the treatment and adherence support according to your allocated group. Neither you nor your doctor can decide which group you join. You must be willing to accept whichever treatment group you are allocated to.

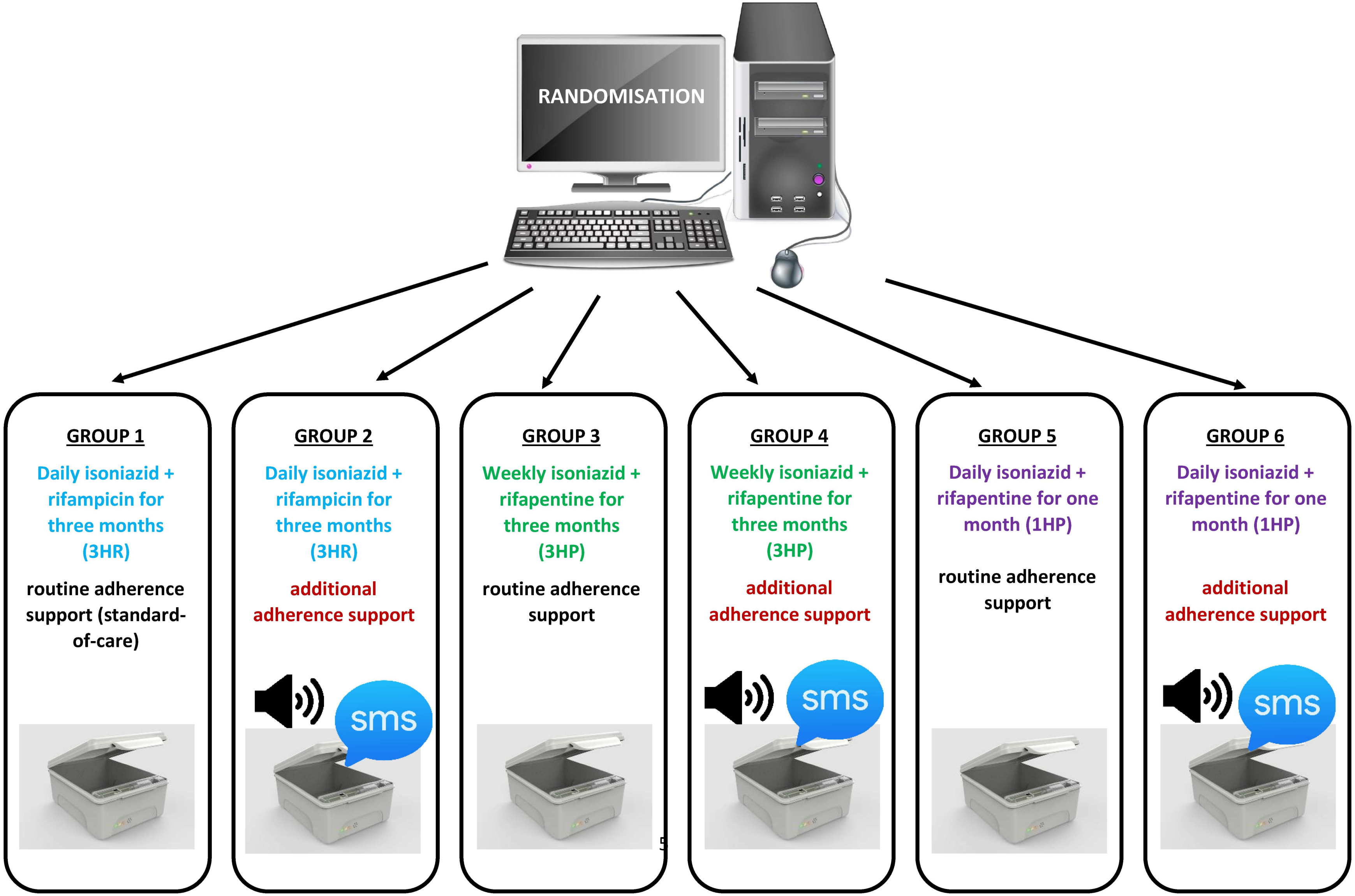
RANDOMISATION
GROUP 1
Daily isoniazid + rifampicin for three months (3HR)
routine adherence support (standard-of-care)
GROUP 2
Daily isoniazid + rifampicin for three months (3HR)
additional adherence support
sms
GROUP 3
Weekly isoniazid + rifapentine for three months (3HP)
routine adherence support
GROUP 4
Weekly isoniazid + rifapentine for three months (3HP)
additional adherence support
sms
GROUP 5
Daily isoniazid + rifapentine for one month (1HP)
routine adherence support
GROUP 6
Daily isoniazid + rifapentine for one month (1HP)
additional adherence support
sms

What will happen to me during the study?

Before randomisation, your doctor will again check for signs of active TB and perform a urine pregnancy test. As usual practice, your doctor will provide you with information about latent TB and why it is necessary to take pills as prescribed. As part of the study, you will be given an electronic pill box that automatically records each opening of the box and sends data to the research team via the internet.

If you are allocated to a group with additional adherence support (Groups 2, 4 and 6) you will be asked to set a reminder using the pill box. This can send an SMS message to your phone or sound an alarm at scheduled times or when the box was not opened in a day. You will also be given adherence support materials such as a video animation to watch. Your study team will collect your phone number and will share with selected members of the UCL study team in order to send an SMS message. The study team at UCL will organise the reminders to be sent.

The electronic pill box will collect data on when you open the box and this will be accessed by the team at UCL.

Once you start treatment, you will be required to visit the clinic at week 2 for blood tests to check side effects in accordance with usual care. Additionally, you will have a consultation every month until completion of treatment to assess your health, including checks for signs of active TB and side effects. You will be requested to bring the pill box to check remaining tablets. Blood tests may be performed if your doctor finds it necessary to check liver problems or other side effects. Pregnancy tests will be done at every visit for women of who are able to become pregnant. After completion of treatment, you will receive a phone call every 4 weeks until 20 weeks after start of treatment to check for signs of active TB and side effects.

If you agree to take part in the optional /or substudies you will be required to complete additional questionnaires at every visit.

8. What are the possible disadvantages and risks of taking part?

As with the standard treatment for LTBI, there is a risk of side effects such as liver problems, allergic reactions and flu-like symptoms. The drugs in this study should not be used during pregnancy, therefore women and their partners must use contraception. For women who are able to become pregnant, pregnancy tests will be repeated throughout the study and treatment will be stopped immediately if a participant becomes pregnant.

9. More information about taking part

Do I have to take part in the RID-TB-Treat study?

No, it is up to you to decide whether to take part or not. ,

If you decide not to take part in this study you are likely to receive the standard treatment for LTBI which is antibiotics for three months (daily) and usual care to check and support your adherence. A decision not to take part at any time will not affect the standard of care you receive.

Will I get back any travel costs?

There will not be any reimbursement for travel costs because this study will *not* require you to visit the hospital more times than if you were being treated in the usual way for latent TB.

Can I stop taking part after I've joined the study?

You can stop taking part in all of this study, or any part of it, at any time and without giving a reason. However, you must talk to your study doctor or nurse first. They can advise you about any concerns you may have.

If you decide to stop taking your study treatment, we will need to continue collecting information about you. This is important because it helps us to ensure that the results of the study are reliable.

If you stop taking part in this study you are likely to receive the standard treatment. A decision to stop taking part at any time will not affect the standard of care you receive.

How will my personal information be used?

University College London (UCL) is the sponsor for this study, based in the United Kingdom. University College London (UCL) will be using information from you and your medical records in order to undertake this study and will act as data controller for this study. University College London (UCL) will be responsible for looking after your information and using it properly, and will keep identifiable information about you for 25 years after the study has finished.

Your rights to access, change or move your information are limited, as we need to manage your information in specific ways in order for the research to be reliable and accurate. If you withdraw from the study, we will keep the information about you that we have already obtained. To safeguard your rights, we will use the minimum personally identifiable information possible.

You can find out more about how we use your information at www.ctu.mrc.ac.uk/general/privacy-policy

How will my data be stored and collected?

Your hospital will collect information from you and your medical records for this research study in accordance with our instructions. Your hospital will use your name, NHS number and contact details to: contact you about the research study, make sure that relevant information about the study is recorded for your care, and to oversee the quality of the study.

UCL will collect information about you for this research study from your hospital, NHS Digital and Public Health England (PHE). This information will include your name, postcode and NHS number and health information. Health information is regarded as a special category of information as defined by the General Data Protection Regulation (GDPR). We will use this information to check whether you develop active TB or become pregnant up until one year after study treatment (https://digital.nhs.uk/).

Where information could identify you, the information will be held securely with strict arrangements about who can access the information.

Future research

When you agree to take part in a research study, the information about your health and care may be provided to researchers running other research studies in this and other organisations. They may be universities, NHS institutions or companies involved in health and care research in this country or abroad. Your information will only be used by organisations and researchers to conduct research in accordance with relevant legislation, ethics and NHS research policy requirements.

We won't share information that can identify you with others. The information will only be used for the purpose of health and care research, and cannot be used to contact you or

to affect your care. It will not be used to make decisions about future services available to you, such as insurance. If there is a risk that you can be identified, your data will only be used in research that has been independently reviewed by an ethics committee.

What will happen to the results of the RID-TB:Treat study?

When the study is completed, we will publish a summary of the results on the website of the MRC CTU at UCL: http://www.ctu.mrc.ac.uk/

We will also publish the results in a medical journal, so that other doctors can see them. You can ask your doctor for a copy of any publication. Your identity and any personal details will be kept confidential. No named information about you will be published in any report of this study.

Who is organising and funding the study?

This study is organised by the MRC CTU at UCL on behalf of The Whittington NHS Trust. The MRC CTU at UCL has run trials for many years. The study coordination, data collection and analysis and administration will be provided by the MRC CTU at UCL. You can find out more about us at www.ctu.mrc.ac.uk

Your doctor is not receiving any money or other payment for asking you to be part of the study. UCL has overall responsibility for the conduct of the study. We are responsible for ensuring the study is carried out ethically and in the best interests of the study participants. A patient representative has been involved in the design of this study and in writing of this information.

Who has reviewed the RID-TB-Treat study?

The study has been reviewed by scientists. It has been approved by the Research Ethics Committee of London Riverside and the National Institute of Health Research (NIHR) who are the funders of the study. It has been authorised by the Medicines and Healthcare products Regulatory Agency (MHRA), as well as by the NHS Health Research Authority (HRA) and the hospital’s Research and Development Office.

What if new information becomes available during the course of the study?

Sometimes during a study, new information becomes available about the medicines and procedures that are being studied. If this happens your doctor will tell you about it and discuss with you whether you want to continue the study. If you decide to stop taking part, your doctor will arrange for your care to continue outside of the study.

Your doctor might also suggest that it is in your best interest to stop taking part in the study. They will explain the reasons and arrange for your care to continue outside the study.

What happens if the RID-TB-Treat study stops early?

Very occasionally a study is stopped early. If it happens, the reasons will be explained to you and your doctor will arrange for your care to continue outside of the study.

What if something goes wrong for me?

If you have any concerns about the way you have been approached or treated during the study, please talk to your study doctor or nurse. If you are still unhappy, or if you wish to complain, please use the normal NHS complaints process.

If you are harmed by taking part in the study, or if you are harmed because of someone’s negligence, then you may be able to take legal action. The study is covered by the sponsor’s

insurance. Further information can be obtained from the study team on request.

10. Contacts for further information

If you want further information about the RIDTB-Treat study, contact your study doctor or nurse (see below).

[Insert address and telephone number of study doctor and/or nurse]

Thank you for taking the time to consider taking part in this study.

RID-TB:Treat protocol version 3.0, 23-Oct-2020

Rangaka MX, *et al. BMJ Open* 2022; 12:e057717. doi: 10.1136/bmjopen-2021-057717
