# Supplementary material for: Evaluating the effect of short-course rifapentine-based regimens with or without enhanced behaviour-targeted treatment support on adherence and completion of treatment for latent tuberculosis infection among adults in the UK (RID-TB: Treat): protocol for an open-label, multicentre, randomised controlled trial
Source: BMJ Open. 2022 Sep 6;12(9):e057717. doi: 10.1136/bmjopen-2021-057717 (PMC9454004; doi:10.1136/bmjopen-2021-057717)
Supplement: Supplementary data [file bmjopen-2021-057717supp002.pdf]

(To be presented on local headed paper)

RID-TB:Treat Informed Consent Form
Version 1.0, 26-Aug-2020
IRAS ID: 282304

Centre Name & Number	
Patient ID Number	
Name of Researcher	

#		Initial to Agree	
1	I have read and understood the information sheet for the RID-TB:Treat research study **[Version 1.0, 26-Aug-2020]** and have been given a copy to keep. I have had the chance to ask questions about the project and discuss it with the study staff. I have received answers to all of my questions.		
2	I understand that my medical notes may be looked at by individuals from the Medical Research Council (MRC) Clinical Trials Unit (CTU), or from regulatory authorities where it is relevant to my taking part in this research. I give permission for these individuals to access my records. I understand that my confidentiality will be maintained.		
3	I understand that participation in this trial is voluntary and that I am free to withdraw from the trial at any time, without giving any reason and without my medical care or legal rights being affected.		
4	I understand that I may not benefit directly by participating in this study but that the research may help people with this condition in the future.		
5	In order to follow-up on my health status after my participation in the trial, I give permission for my personal details (such as NHS number, name and date of birth) to be used to obtain information about my health status from records held by NHS Digital, Public Health England, the National TB register, or any applicable national or NHS information system. I understand that this information may be obtained about me during the study and after (up to 25 years).		
6	**I agree to take part in the RID-TB:Treat study.**		
**Optional Items:** *If you wish to give permission, put your initials in the 'Yes' box. If you do not wish to give permission, put your initials in the 'No' box. If you do not agree to any of the following items, you can still take part in the main study.*		**Yes**	**No**
7	I agree for my GP to be informed of my participation in the research study.		
8	I agree to participate in the Behavioural Sub-study and to complete the questionnaires.		
9	I agree to participate in the Health Economics Sub-study and to complete the questionnaires.		

Signatures

Name of Participant (BLOCK CAPITALS)	Date (Day/month/year)	Signature (or thumbprint)
Name of Witness (BLOCK CAPITALS)	Date (Day/month/year)	Signature (if thumbprint used above)
Name of person taking consent (BLOCK CAPITALS)	Date (Day/month/year)	Signature

IMPORTANT: Signed original to be kept in the Investigator Site File
One copy to be given to the participant
One copy to be kept with the participant's medical notes
