# Supplementary material for: Evaluating the effect of short-course rifapentine-based regimens with or without enhanced behaviour-targeted treatment support on adherence and completion of treatment for latent tuberculosis infection among adults in the UK (RID-TB: Treat): protocol for an open-label, multicentre, randomised controlled trial
Source: BMJ Open. 2022 Sep 6;12(9):e057717. doi: 10.1136/bmjopen-2021-057717 (PMC9454004; doi:10.1136/bmjopen-2021-057717)
Supplement: Author's manuscript [file bmjopen-2021-057717.draft_revisions.pdf]

BMJ Open is committed to open peer review. As part of this commitment we make the peer review history of every article we publish publicly available.

When an article is published we post the peer reviewers' comments and the authors' responses online. We also post the versions of the paper that were used during peer review. These are the versions that the peer review comments apply to.

The versions of the paper that follow are the versions that were submitted during the peer review process. They are not the versions of record or the final published versions. They should not be cited or distributed as the published version of this manuscript.

BMJ Open is an open access journal and the full, final, typeset and author-corrected version of record of the manuscript is available on our site with no access controls, subscription charges or pay-per-view fees (http://bmjopen.bmj.com).

If you have any questions on BMJ Open's open peer review process please email info.bmjopen@bmj.com

BMJ Open

An open-label, multi-centre, randomised controlled trial to evaluate the effect of short-course rifapentine-based regimens with or without enhanced behaviour-targeted treatment support on adherence and completion of treatment for latent TB infection among adults in the UK (RID-TB: Treat): a study protocol

Journal:	*BMJ Open*
Manuscript ID	bmjopen-2021-057717
Article Type:	Protocol
Date Submitted by the Author:	27-Sep-2021
Complete List of Authors:	Rangaka , Molebogeng ; University College London; University of Cape Town<br>Hamada, Yohhei; University College London, Institute for Global Health<br>Ghanouni, Alex; UCL School of Pharmacy<br>Layton, Charlotte; MRC Clinical Trials Unit at UCL<br>Duong, Trinh; MRC Clinical Trials Unit at UCL<br>Bern, Henry; MRC Clinical Trials Unit at UCL<br>Calvert, Joanna ; MRC Clinical Trials Unit at UCL<br>Francis, Marie; University College London<br>Clarke, Amy; University College London, School of Pharmacy<br>Hack, Vanessa ; University College London, Institute for Global Health<br>Owen-Powell, Ellen; MRC Clinical Trials Unit at UCL<br>Surey, Julian; University College London<br>Sanders, Karen; MRC Clinical Trials Unit at UCL<br>Booth, Helen; University College London Hospitals NHS Foundation Trust<br>Crook, Angela; University College London Institute of Clinical Trials and Methodology, MRC Clinical Trials Unit<br>Griffiths, Chris; Barts and The London School of Medicine and Dentistry<br>Horne, Robert; UCL School of Pharmacy<br>Kunst, Heinke; Queen Mary University of London, Department of Respiratory Medicine<br>Lipman, Marc; Royal Free London Hospital NHS Foundation Trust; University College London<br>Mandelbaum, Mike; TB Alert<br>White, Peter; Imperial College, School of Public Health; Public Health England, Modelling and Economics Unit<br>Zenner, Dominik; University College London Institute for Global Health; Queen Mary University of London, Wolfson Institute for Population Health Barts and the London School of Medicine and Dentistry<br>Abubakar, Ibrahim; University College London
**Primary Subject Heading**:	
Secondary Subject Heading:	

Keywords:	Tuberculosis < INFECTIOUS DISEASES, Clinical trials < THERAPEUTICS, PREVENTIVE MEDICINE

SCHOLARONE™
Manuscripts

An open-label, multi-centre, randomised controlled trial to evaluate the effect of short-course rifapentine-based regimens with or without enhanced behaviour-targeted treatment support on adherence and completion of treatment for latent TB infection among adults in the UK (RID-TB: Treat): a study protocol

Molebogeng X. Rangaka[1,2], Yohhei Hamada[1], Trinh Duong[3], Henry Bern[3], Joanna Calvert[3], Marie Francis[1], Amy Louise Clarke[4], Alex Ghanouni[4], Charlotte Layton[3], Vanessa Hack[1], Ellen Owen-Powell[3], Julian Surey[1], Karen Sanders[3], Helen L. Booth[5], Angela M. Crook[3], Chris Griffiths[6], Rob Horne[4], Heinke Kunst[7], Marc Lipman[8,9], Mike Mandelbaum[10], Peter J. White[11, 12], Dominik Zenner[1],[6], Ibrahim Abubakar[1]

[1] Institute for Global Health, University College London, London, UK

[2] University of Cape Town, Cape Town, South Africa

[3] MRC Clinical Trials Unit at UCL, Institute of Clinical Trials and Methodology, London, UK

[4] Centre for Behavioural Medicine, UCL School of Pharmacy, London, UK

[5] North Central London Tuberculosis Service, Whittington Health NHS Trust and University College London Hospitals NHS Foundation Trust, UK London, UK

[6] Wolfson Institute for Population Health Barts and the London School of Medicine and Dentistry, Queen Mary University of London, UK

[7] Blizard Institute, Barts and The London School of Medicine and Dentistry, Queen Mary University of London, UK

[8] Royal Free London Hospital NHS Foundation Trust, London, UK

[9] UCL Respiratory, Division of Medicine, University College London, London, UK.

[10] TB Alert, London, UK

[11] MRC Centre for Global Infectious Disease Analysis, Imperial College London, London, UK

[12] Modelling and Economics Unit, National Infection Service, Public Health England, London, UK

Corresponding author

Molebogeng X Rangaka

Institute for Global Health, University College

30 Guilford Street, London WC1N 1EH

For peer review only - http://bmjopen.bmj.com/site/about/guidelines.xhtml

Email: l.rangaka@ucl.ac.uk

Phone: +44-7552-526872

Keywords: clinical trial; LTBI; medication adherence; rifapentine; tuberculosis

For peer review only

Abstract

Introduction

The successful scale-up of a latent tuberculosis infection (LTBI) testing and treatment programme is essential to achieve TB elimination. However, poor adherence compromises its therapeutic effectiveness. Novel rifapentine-based regimens and treatment support based on behavioural science theory may improve treatment adherence and completion.

Methods and analysis

A pragmatic multi-centre open-label randomised controlled trial to assess the effect of novel short-course rifapentine-based regimens for TB prevention and additional theory-based treatment support on treatment adherence against standard-of-care. Participants aged between 16 and 65, with a positive LTBI test who are eligible to start TB preventive therapy will be recruited. A total of 920 participants will be randomized to one of the six arms with an allocation ratio of 5:5:6:6:6:6: (1) daily isoniazid + rifampicin for three months (3HR), routine treatment support (control arm); (2) 3HR, additional treatment support; (3) weekly isoniazid + rifapentine for three months (3HP), routine treatment support; (4) weekly 3HP, additional treatment support; (5) daily isoniazid + rifapentine for one month (1HP), routine treatment support; (6) daily 1HP, additional treatment support. Additional treatment support comprises reminders using an electronic pill box, short animation, and leaflets based on the Perceptions and Practicalities Approach. The primary outcome is adequate treatment adherence, defined as taking $\geq$ 90% of allocated doses within the pre-specified treatment period, measured by electronic pill boxes. Secondary outcomes include safety and TB incidence within 12 months.

We will also conduct process evaluation of the trial interventions and assess intervention acceptability and fidelity and mechanisms for effect and estimate the cost-effectiveness of novel regimens. The protocol was developed with Patient and Public Involvement, which will continue throughout the trial.

Ethics and dissemination

Ethics approval has been obtained from The NHS Health Research Authority (20/LO/1097). We will share the results in peer-reviewed journals.

For peer review only - http://bmjopen.bmj.com/site/about/guidelines.xhtml

Trial registration number EudraCT 2020-004444-29

For peer review only - http://bmjopen.bmj.com/site/about/guidelines.xhtml

For peer review only

Article summary

Strengths and limitations of this study

- This is the first trial to assess the effect of two rifapentine-based regimens compared to the standard 3-month daily rifampicin plus isoniazid, and the effect of additional treatment support compared to routine support, on LTBI treatment adherence. The intervention was developed through a robust iterative process including patient and public involvement, semistructured interviews and questionnaires with patients and clinicians.
- We will perform process evaluation of the trial interventions, including assessment of intervention acceptability and fidelity, and economic evaluation, which will provide additional evidence to inform treatment options and treatment support.
- The trial will involve six arms because of the potential interaction between treatment regimens and treatment support. The trial is powered to evaluate novel rifapentine-based regimens compared to the standard daily rifampicin plus isoniazid (3HR) and the effect of additional treatment support compared to routine support; however, it does not have sufficient power to evaluate all possible comparisons such as 3-month weekly rifapentine plus isoniazid vs 1-month daily rifapentine plus isoniazid.
- The trial will be conducted in England largely in migrant populations eligible for the LTBI screening programme and contacts of TB patients and thus limiting generalisability to these populations and similar settings.
- Adherence will be measured using electronic pill boxes in all arms while reminders will be activated only in arms with additional treatment support; however, this may impact adherence in control groups.

For peer review only - http://bmjopen.bmj.com/site/about/guidelines.xhtml

INTRODUCTION

Successful implementation of screening and treatment for latent tuberculosis infection (LTBI) is critical to further reduce TB incidence globally and achieve TB elimination in low TB incidence countries.(1) A recent call to action issued by the World Health Organization urged for accelerating the scale-up of treatment of LTBI, particularly to mitigate the negative impact from the disruption of TB services caused by the pandemic of COVID-19.(2)

Tuberculosis (TB) in England disproportionately affects underserved communities, such as migrants and homeless people, who consequently experience higher disease burden and worse clinical outcomes. Consequently, in England, LTBI screening and treatment for high risk groups such as new migrants from high TB incidence countries is recognized as an essential strategy to achieve TB elimination.(3) Contact tracing, including testing and treatment of LTBI among contacts, is another essential component of the TB strategy for England.(3)

Achieving optimal treatment adherence and completion is essential to ensure the efficacy of treatment for LTBI and to achieve commensurate reductions in TB incidence. Standard therapeutic options in the UK include 3-months of self-administered daily isoniazid/rifampicin and 6-months of daily isoniazid; the former regimen is often prescribed because of the availability of fixed-dose formulations and its shorter duration. Whilst these regimen are efficacious in preventing disease, their effectiveness is limited by low treatment adherence and completion rates.(4, 5) According to data from England in migrants whose treatment outcome is known, 75% completed LTBI treatment between 2019 and 2020.(6, 7) The proportion of people who completed treatment varied by Clinical Commission Group (CCG), which was less than 70% in several CCGs.(8)

People with LTBI may need additional support to adhere to effective treatments. Treatment non-adherence can be intentional or unintentional, and is driven by a person's motivation and ability to take medicine as prescribed, respectively.(9) Motivation is influenced by our perceptions (e.g. beliefs and preferences) and ability is determined by practical factors (e.g. internal capacity and resource).(9)

For peer review only - http://bmjopen.bmj.com/site/about/guidelines.xhtml

These principles are operationalised as part of the Perceptions and Practicalities approach to supporting adherence (PAPA) and are applied in NICE guidelines.(10) The Necessity and Concerns Framework (NCF) further explains how patients motivation to engage with treatment is based on their perceived necessity for, and concerns about the treatment.(11) Necessity beliefs are influenced by perceptions of the health threat (e.g LTBI) and interpretation of symptoms. The asymptomatic nature of LTBI may negatively impact necessity beliefs, and heighten treatment concerns. As such, intervention to support treatment adherence in people with LTBI will likely be more effective if they address patient beliefs and concerns around treatment, in addition to removing practical barriers.

The need to understand perceptual and practical barriers to treatment adherence, and the potential of advancing technology and drug regimens in the NHS has been highlighted. Some Mobile/digital technology (mHealth) has been shown to improve adherence in TB disease studies. A recent study in China found electronic reminders, using specially designed electronic medication monitors, improved treatment adherence in such TB patients, but multiple two-way daily text messaging reminders, didactic in nature, did not.(12) Most of the evidence available is on TB disease with little research on mHealth interventions to improve LTBI treatment adherence.(13, 14) Another call to action issued by the World Health Organization suggested TB preventive treatment programmes should consider communication technologies for medication adherence support.(15) The evidence on mHealth interventions for LTBI treatment would contribute to their global scale-up.

Another approach to promote better treatment adherence and completion is to decrease the complexity of current LTBI regimens. A regimen that is given once weekly may result in better treatment completion than the current daily 3-month regimen. A randomised controlled trial demonstrated that a new regimen of 12 doses of weekly rifapentine and isoniazid (3HP) delivered through direct observation (i.e. with patients being supervised taking each dose) is non-inferior to 9 months of daily isoniazid.(16) Our network meta-analysis suggests that 3HP has similar efficacy to the UK standard-of-care of a 12-week, daily isoniazid/rifampicin regimen (3HR).(17) Furthermore, a recent trial in people living with HIV (23% with LTBI as demonstrated by a positive TST and/or IGRA result)

For peer review only - http://bmjopen.bmj.com/site/about/guidelines.xhtml

demonstrated non-inferiority of daily one-month rifapentine plus isoniazid (1HP) compared to 9 months of daily isoniazid.(18) The one-month regimen resulted in better adherence and fewer serious adverse events. Based on this study, and by extrapolating to HIV-negative individuals, newly published WHO guidelines recommend this regimen regardless of HIV status. However, there is no published evaluation of whether these more expensive rifapentine-based regimens lead to better treatment completion than the current daily administered UK standard-of-care. In particular, evidence is limited on the use of 3HP with patient self-administration and no study has compared its completion with 3HR.

To develop tools to reduce TB rates, we need to evaluate advancing technology and drug regimens, but also understand the barriers and enablers of adherence.(3) To date, adherence interventions have predominantly focused on removing practical barriers to adherence (e.g reminder of shortening the drug regimen). However, such approaches applied in isolation ignore patient beliefs. LTBI is asymptomatic which means patients might have a disconnect between medical advice and their perceived need for treatment.(19) NICE guidelines recommend a Perceptions and Practicalities Approach (PAPA) to adherence support, whereby beliefs (necessity and concerns) are elicited and addressed in addition to practical barriers.(10, 11)

We previously conducted the HALT-LTBI study, a pilot study assessing the safety and treatment completion of 3HP compared to standard care.(20) The HALT-LTBI demonstrated the feasibility of recruiting LTBI patients to such a trial; no serious adverse events defined as grade 3 or more were reported, supporting the safety of rifapentine and isoniazid regimens in individuals eligible for LTBI treatment in the UK. 78% and 68% of participants completed treatment in the experimental and standard-of-care arms, respectively, but the pilot was not powered to detect differences in treatment completion. Thus, we will conduct a fully powered trial to compare treatment adherence and adverse events of novel 3HP and 1HP regimens compared with 3HR and to assess the effect of additional treatment support in participants given each regimen.

Objectives

For peer review only - http://bmjopen.bmj.com/site/about/guidelines.xhtml

The primary objective of this trial is to assess the effect of novel rifapentine-based regimens (3HP or 1HP) compared to the standard 90-dose daily rifampicin plus isoniazid (3HR), and the effect of additional treatment support compared to routine support, on LTBI treatment adherence.

The secondary objectives are: 1) to evaluate the effect of LTBI treatment and additional treatment support using alternate measures of adherence outcome; and 2) to compare the frequency of adverse events whilst on treatment for LTBI, and development of TB within 12 months following treatment. Additionally, we will evaluate the process of delivering the adherence intervention and examine intervention fidelity and acceptability as well as the cost-effectiveness of different treatment options and/or additional treatment support.

METHOD AND ANALYSIS

Trial design

A multi-centre open-label randomised controlled trial with the following six parallel groups (Figure 1):

ARM 1- Daily isoniazid + rifampicin for three months (3HR), routine treatment support (SOC; control arm)

ARM 2- Daily 3HR, additional treatment support

ARM 3- Weekly isoniazid + rifapentine for three months (3HP), routine treatment support

ARM 4- Weekly 3HP, additional treatment support

ARM 5- Daily isoniazid + rifapentine for one month (1HP), routine treatment support

ARM 6- Daily 1HP, additional treatment support

A factorial design was not chosen for several reasons. Firstly, it is anticipated that there will be an interaction between type of regimen and treatment support; additional treatment support is likely to confer a smaller benefit with 3HP/1HP compared to 3HR. Secondly, the power to detect the effect of an intervention would be reduced if the effect of the second intervention is greater than expected.

For peer review only - http://bmjopen.bmj.com/site/about/guidelines.xhtml

Study setting

The trial will recruit from secondary care sites that provide LTBI treatment in England, UK. RID-TB: Treat is part of a 5-year programme of work (RID-TB) which is funded by the National Institute for Health Research (NIHR) (RP-PG-0217-20009 https://dev.fundingawards.nihr.ac.uk/award/RP-PG-0217-20009).

Study population

The trial will enrol populations who are eligible for treatment for LTBI according to the national guidance. We envisage that the majority of individuals eligible for this are contacts of persons diagnosed with TB disease, and/or migrants eligible for the national LTBI screening programme ref. The LTBI migrant screening programme includes migrants who are aged 16 to 35 years, entered the UK from a high incidence country (≥150/100,000) or Sub-Saharan Africa within the last five years and had been previously living in that high incidence country for six months or longer.(21) Inclusion and exclusion criteria are shown in Box 1.

Participants will be identified from secondary care settings in the UK where persons eligible for treatment for LTBI are managed. Participants will be recruited individually, but if any participants share a household, they will be allocated to the same arm as the first person recruited from that household (effectively resulting in randomisation by household).

Treatment

Participants who are randomized to arms 1 and 2 will receive the standard of care regimen: rifampicin plus isoniazid once daily for 90 doses (3HR).

Participants who are randomized to arms 3 and 4 will receive rifapentine plus isoniazid once weekly for 12 doses (3HP) and those who are randomized to arms 5 and 6 will receive rifapentine plus isoniazid once daily for 28 doses (1HP).

For peer review only - http://bmjopen.bmj.com/site/about/guidelines.xhtml

In order to account for missed doses and interruption of treatment due to adverse events, participants given 3HR or 3HP will have 16 weeks and those given 1HP will have 6 weeks to complete treatment. In the study by Swindells et al, participants were given 8 weeks to complete 1HP.(18) We have chosen 6 weeks to make the period proportionally similar to that for 3HR and 3HP. Clinicians will assess the need for treatment extension based on the assessment of adherence and review of reasons for non-adherence but should not extend beyond recommended grace periods.

In all arms, participants will receive vitamin B6 (pyridoxine). The dosages of study drugs are shown in Table 1.

Treatment support

Routine treatment support

Participants allocated to arms 1, 3, and 5 will receive routine treatment support. Participants will be given information about treatment for LTBI including expected adverse events and the importance of adherence, according to local practice. Adherence will be reviewed at each follow-up visit or remote consultation via self-reporting and/or pill count and discussed with the participant. An electronic pill monitor box, Wisepill EvriMed1000 (Wisepill, Somerset West, South Africa)(22) will collect the date and time of each opening to collect information on adherence. However, it will be set to silent mode and not be used as an adherence reminder tool.

Intervention

Participants assigned to arms 2, 4, and 6 will receive a PAPA-based intervention designed to provide additional treatment support (i.e. in addition to routine treatment support).(11) Specifically, the intervention will consist of an animation which will 1) provide a rationale for treatment necessity and help people understand how LTBI treatment can help them to achieve a health goal that is important to them, 2) address common concerns about LTBI treatment and 3) address practical barriers to treatment (e.g. anchoring treatment to daily activities). The animation will be supported by a leaflet

For peer review only - http://bmjopen.bmj.com/site/about/guidelines.xhtml

that cover misperceptions about LTBI testing and treatment, and other frequently asked questions. Participants will also be asked to set reminders using an electronic pill monitor box (Wisepill EvriMed). The electronic pill box allows two modes of reminders: audio alarm from the box or text-message to participants' mobile phones.

The reminder can be set at pre-specified times and can also be activated to send a reminder when the pill box is not opened. Site staff will discuss options with each participant and set reminders according to their preferences. Participants can opt not to receive reminders before or at the time of intended medication intake. However, they will still be reminded when the box is not opened within a pre-specified time in a day and they will receive a supportive text message automatically sent by the pill box. The mode of reminder can be further adjusted during the course of treatment as necessary upon discussion with a clinician. The pill box will electronically collect the date and time of each opening.

Study assessment and follow-up

Screening, randomisation and baseline assessment

Randomisation and baseline assessment will occur on the same day (Week 0). In some cases, this may also be the same day as Screening. Following informed consent procedures, participants will be screened for eligibility. A TB symptom screen and urine pregnancy test will be carried out, and data on the participant's TB risk group category will be collected. Demographic and medical history information will be collected. We will check the results of clinical, laboratory, and radiological assessments performed under routine care before entry to the trial to confirm eligibility. A TB symptom screen and urine pregnancy test will be repeated at the randomization/baseline visits unless the screening and randomisation visits occur on the same day.

Assessment of Adherence

Assessment of adherence will be primarily measured using the Wisepill, which collects the date and time of each opening. Adherence will also be measured through self-reporting and pill count under routine care either at physical clinic visits or remote consultations as per the local standard. Attending

For peer review only - http://bmjopen.bmj.com/site/about/guidelines.xhtml

clinicians will count the number of remaining tablets. The difference between the number of tablets dispensed and the number returned will be calculated.

Clinical assessment during follow-up

As per usual practice, liver function tests (hepatic transaminases, ALT/AST, and total bilirubin) will be performed at week 2 for all participants. Afterwards, liver function tests will be performed at weeks 4, 8, 12, and 16 while on treatment and at completion, or at other times if deemed necessary by attending clinicians (e.g. abnormality in preceding tests, new onset of symptoms suggesting potential liver toxicity). These tests should be performed at any time during the treatment and post-treatment phase if the participant exhibits symptoms or signs of drug-induced liver injury (DILI).

Symptoms and signs of adverse events expected withstudy drugs, including: anorexia, nausea, vomiting, fatigue, weakness, jaundice, rash, peripheral neuropathy, bruising will be clinically assessed at every visit. Participants who already completed treatment and have no scheduled visits will be given a phone call at week 8, 12, 16 and 20 to check adverse events and TB signs and symptoms since the last dose.

At every physical visit or remote consultation, we will review symptoms and signs of TB disease as well as review concomitant medications using a brief questionnaire. There will be no formal study visits after completion of treatment.

Outcomes

Primary Outcome

The primary outcome is adequate treatment adherence, defined as taking ≥90% of allocated doses within the allowable time-frame from randomisation (binary outcome). For the primary analyses, treatment adherence is measured using an electronic monitor box

Secondary Outcomes

The secondary outcome measures are:

For peer review only - http://bmjopen.bmj.com/site/about/guidelines.xhtml

- Effectiveness: (1) Proportion of allocated doses missed over the treatment period (measured using monitor box); (2) Proportion of allocated pills missed over the treatment period (measured using pill counts); (3) Taking at least 90% of doses and pills over the treatment period (binary outcome assessed using both monitor box and pill counts); (4) Early study treatment discontinuation for any reason
- Safety: (1) Permanently stop study treatment due to drug-related adverse events (i.e. adverse reactions) ; (2) Experience Grade ≥3 adverse events; (3) Develop TB disease within 12 months.

Sample size

The six-arm design allows evaluation of:

- the effect of the novel treatment regimens (3HP and 1HP) versus standard-of-care regimen (3HR), under routine treatment support
- the effect of additional treatment support vs routine treatment support for each individual regimen

A total of 920 participants are to be recruited. This provides 80% power for each of the following comparisons:

- Arm 3 vs Arm 1- ie 3HP + routine treatment support vs 3HR + routine treatment support
- Arm 5 vs Arm 1- ie 1HP + routine treatment support vs 3HR + routine treatment support
- Arm 2 vs Arm 1- ie 3HR + additional treatment support vs 3HR + routine treatment support
- Arm 4 vs Arm 3- ie 3HP + additional treatment support vs 3HP + routine treatment support
- Arm 6 vs Arm 5- ie 1HP + additional treatment support vs 1HP + routine treatment support

The power calculations assume the following:

- 70% adherence rate in Arm 1

For peer review only

For peer review only - http://bmjopen.bmj.com/site/about/guidelines.xhtml

- 3HP and 1HP improve adherence rate by 15% (absolute difference) compared to 3HR, respectively, with routine treatment support(18, 23, 24)
- Compared to routine treatment support, additional treatment support improves adherence rate by 15% for 3HR, and 10% for 3HP and 1HP, respectively(12)
- 2-sided alpha 5% (see below for type I error considerations)
- average number of participants enrolled per household is 2, taking into account the average household size in UK.(25)
- intra-class correlation (ICC) within a household is 0.1

The 70% adherence rate assumed for Arm 1 is based on the 77% LTBI treatment completion rate reported from the Public Health England LTBI testing and treatment database for 2018.(26)

Randomisation and allocation

Participants will be randomised centrally using a computerised algorithm developed and maintained by the Medical Research Council Clinical Trials Unit at University College London (MRC-CTU). To randomise a participant, the information contained on a completed Randomisation Form will be entered into the secure online trial database by trial team members at the site who have been trained and authorised to randomise by the MRC-CTU. The database will automatically check for eligibility. Only those who meet all eligibility criteria will be able to be randomised. Randomisation will be performed using minimisation with an additional random element, to be balanced with respect to centre and TB exposure risk group.

Blinding

This is an open-label trial. Blinding of participants and care providers to the allocation group is not relevant since the primary objective of this trial to examine the effect of shorter or weekly regimens and additional treatment support on treatment adherence.

Data collection methods and management

For peer review only

Adherence data will be collected through the Wisepill monitor box. Demographic and clinical information will be collected through clinical consultation and recorded in relevant case report forms (CRFs). Development of TB within 12 months after starting treatment and outcomes of pregnancy that is found after enrolment will be collected using records held by NHS Digital, Public Health England, and/or the National TB register.

Statistical methods

The estimands for the primary analyses are defined in Table 2. The primary analyses will compare the proportion of participants with adequate adherence between arms using the following approach:

a) Arm 3 vs Arm 1- ie 3HP + routine treatment support vs 3HR + routine treatment support
b) Arm 5 vs Arm 1- ie 1HP + routine treatment support vs 3HR + routine treatment support
c) Arm 2 vs Arm 1- ie 3HR + additional treatment support vs 3HR + routine treatment support

If comparison (a) shows 3HP improves adherence compared to 3HR, then additional treatment support will be formally tested for 3HP by comparing Arm 4 vs Arm 3 – ie 3HP + additional treatment support vs 3HP + routine treatment support; otherwise, the adherence rates will be compared between these arms as exploratory analyses. Additional treatment support will be similarly assessed for 1HP.

All randomised patients will be included in the primary analyses, apart from those subsequently found to have had TB disease at baseline but enrolled in error (modified intention-to-treat approach). The risk ratio (with 95% confidence interval) for adequate treatment adherence comparing the relevant arms will be estimated using log-binomial generalised linear mixed models (GLMMs), allowing for intra-household correlation.

Type I error adjustment for multiple comparisons is not deemed necessary since:

- The research hypotheses corresponding to comparisons (a), (b) and (c) are considered sufficiently distinct.(27-29)
- The effect of additional treatment support versus routine support is being evaluated in non-overlapping populations for 3HR, 3HP and 1HP, respectively.

For peer review only - http://bmjopen.bmj.com/site/about/guidelines.xhtml

- The closed test approach whereby the effect of additional treatment support will only be formally tested for 3HP if there is evidence that 3HP improves adherence compared to 3HR with routine treatment support protects the type I error. This approach will also be used for the assessment of additional treatment support for 1HP.

For participants who have collected all prescriptions but are lost to follow-up before completing treatment, the adherence data until the end of allocated period can still be downloaded remotely from the Wisepill monitor box to ascertain whether adequate treatment adherence is achieved; this data will be included in the primary analyses. In sensitivity analyses, the primary outcome will be imputed for these patients using multiple imputation by chained equations (MICE), with imputation to be conducted separately by study arm. Sensitivity analyses will also be performed assuming no drug intake from the last follow-up visit attended.

Supplementary analyses will consider different definitions of adequate treatment by varying the minimum proportion of doses required to have been taken, and different allowable time-frames for making up missed doses. In addition, other analysis populations will be considered, including intention-to-treat and per protocol (including only participants who commenced their original allocated trial intervention). Planned exploratory subgroup analyses, will examine outcomes in pre-defined subgroups.

Safety reporting

The definitions of the EU Directive 2001/20/EC Article 2 based on the principles of Good Clinical Practice apply to this trial protocol. These definitions are given in Table 3. All Grade 3 or higher adverse events, whether expected or not, will be recorded in the patient's medical notes. All adverse events will be recorded up to week 20. Serious adverse events should be notified to the CTU within 24 hours of the investigator becoming aware of the event from the time of randomisation to the last assessment of adverse events, i.e. week 20. Adverse events will be graded using the DAIDS toxicity grading scale.(30)

For peer review only - http://bmjopen.bmj.com/site/about/guidelines.xhtml

Monitoring and oversight

The trial will be monitored by the MRC-CTU. An Independent Data Monitoring Committee (IDMC) will be formed. The IDMC will review study conduct and safety data regularly. The IDMC will be asked to advise on whether the accumulated data from the trial, together with results from other relevant trials, justify continuing recruitment of further participants. The IDMC will make recommendations to the Trial Steering Committee (TSC) as to whether the trial should continue in its present form.

Process evaluation

The process evaluation will follow MRC guidance using an embedded, mixed-methods evaluation approach in order to assess acceptability, fidelity, and mechanisms of effects of the interventions. It will be conducted by the research team, working closely with the Intervention Development Group and clinicians delivering the trial.

Patient sample

Patients in the full trial sample will be administered validated questionnaires assessing the psychological characteristics that we predict will mediate the effects of the interventions. Questionnaire will be administered during scheduled clinic appointments at baseline (0 weeks), interim (2 weeks) and treatment completion (either 4 or 12 weeks depending of regimen). Baseline measure will include Beliefs about Medicines Questionnaire (BMQ-Specific/BMQ-General), Perceived Sensitivity to Medicines Scale (PSM-5), Brief illness perceptions questionnaire (BIPQ), The Satisfaction with Information about Medicines Scale (SIMS), Hospital Anxiety and Depression Scale (HADS). At follow-up participants will complete the BIPQ and BMQ-Specific, and a measure of self-reported adherence (Medication Adherence Report -5 [MARS-5]) and the Treatment intrusiveness Questionnaire (TIQ). A subset of participants will also be approached for a qualitative assessment of their experiences in the trial. Participants in each intervention arm will be purposively sampled based on their treatment adherence (10 participants per arm: 5 high adherence, 5 low adherence; total 60 interviews; adherence in line with the primary outcome). Measures will consist of brief, semi-structured interviews.

For peer review only - http://bmjopen.bmj.com/site/about/guidelines.xhtml

Staff sample

Healthcare professionals responsible for administering the interventions will be requested to complete a short checklist form following patient randomisation in order to assess intervention fidelity. This will confirm whether each component of the interventions was delivered per protocol. We will also purposively sample 20 service providers to take part in brief, semi-structured interviews (in person or by phone) in order to obtain feedback on the delivery of the intervention and to identify any issues that might enhance delivery in practice. In addition, we will use these interviews to investigate wider contextual issues impacting on delivery. We will also encourage implementing clinicians to report major issues that might compromise intervention delivery during the trial, rather than waiting for a formal interview on trial completion.

Health economic evaluation

This will estimate if changes to LTBI diagnosis and/or treatment are cost-effective from the perspective of the National Health Service, using a health-economic model to synthesise data obtained within the entire RID-TB programme and evidence from other sources. Participants will be asked to complete monthly EQ-5D questionnaires. We will collect information on the costs participants incur in attending appointments within this trial, to allow potential future analysis from a societal perspective.

PATIENT AND PUBLIC INVOLVEMENT (PPI)

The trial was discussed with the charity TB Alert and two community representatives drawn from a migrant charity and a patient previously treated for LTBI. A charity representative and one former patient read versions of the grant proposal and contributed suggestions on study design. At the protocol development stage, the following input was sought from TB Alert: study design, treatment support interventions, Participant Information Sheet and Consent form, patient-facing questionnaires used for behavioural studies.

For peer review only - http://bmjopen.bmj.com/site/about/guidelines.xhtml

During the trial, we will engage with 1) The RID-TB PPI Advisory Group (PPAG) consisting of members recruited via social media accounts, TB nurses, TB patient advocates, ex-patient contacts and voluntary/community organisations and (2) The TB Action Group (TAG) network of people personally affected by TB. We will seek input for: recruitment, patient/public engagement tools, provision of translated materials on LTBI and access to recruitment sites.

ETHICS AND DISSEMINATION

Ethics approval

Ethics approval has been obtained from the Health Research Authority (HRA) in the UK (20/LO/1097). Any further substantial amendments will be submitted and approved by the main Research Ethics Committee and HRA.

Consent

Participants will be screened and consented at approved trial sites that are authorised by the MRC-CTU to carry out the RID-TB: Treat trial. We will provide potential participants with a copy of the Participant Information Sheet. We will obtain written informed consent to enter into the trial and be randomised after explanation of the aims, methods, benefits and potential hazards of the trial before any trial-specific procedures are performed.

Dissemination

We will report findings of the trial through publications in national and international conferences as well as in peer-reviewed journals. Findings will be also disseminated via TB Aler, TB Action Group., social media, and institutional websites. Trial data will be available for sharing by request after the primary publication upon approval by the Trial Management Group.

CONCLUSION

This trial will provide evidence on the effect of novel rifapentine-based treatment regimens for LTBI and enhanced treatment support on treatment adherence. Underpinned by process evaluation and

For peer review only

economic evaluation, this trial will inform treatment for LTBI strategies in the UK. The results of this trial may also be of value in other similar settings, and possibly in low and middle-income countries.

Acknowledgements

We thank the NIHR programme officers, UCL-NIHR Patient and Public Involvement Advisory Group, MRC CTU at UCL Protocol Review Committee, and the independent Programme Steering Committee for their support and inputs during the development of the protocol.

Authors contributions

IA and MXR conceived the study. IA and MXR led the application to secure funding. IA, MXR, TD, YH, HB, JC, MF, ALC, AG, VH, EOP, JS, KS, HLB, AC, CG, RH, MJ, HK, ML, MM, PJW, DZ contributed to the study design. TD and AMC provided statistical oversight. MXR, TD, and YH drafted and revised the manuscript. All authors contributed critical intellectual input and approved the final manuscript.

Funding

The protocol reported in this publication was supported by the NIHR (RP-PG-0217-20009) and will receive support from the NIHR Clinical Research Network (NIHR CRN). Additional funding for patient and public involvement was provided by the UCLH/UCL Biomedical Research Centre Patient & Public Involvement bursary fund. AMC and TD are supported by MRC Grant: MC_UU_12023/27.

Competing interests

None declared.

References

1. Lönnroth K, Migliori GB, Abubakar I, et al. Towards tuberculosis elimination: an action framework for low-incidence countries. European Respiratory Journal. 2015;45:928-52.

For peer review only

2. World Health Organization. Call to Action 2.0: A global drive to scale up TB. https://cdn.who.int/media/docs/default-source/hq-tuberculosis/call-to-action-2.0-a-global-drive-to-scale-up-tb-prevention.pdf?sfvrsn=6f938b38_7&download=trueT_scaleup.pdf?u=f093a7c38a3780cd9504f8d9d&id=135bdee6af&e=09bced52fa Accessed 8 July 2021.

3. Public Health England. Collaborative tuberculosis strategy for England: 2015 to 2020. 2015.

4. Hirsch-Moverman Y, Shrestha-Kuwahara R, Bethel J, et al. Latent tuberculous infection in the United States and Canada: who completes treatment and why? The international journal of tuberculosis and lung disease : the official journal of the International Union against Tuberculosis and Lung Disease. 2015;19:31-8.

5. Saunders MJ, Koh GC, Small AD, et al. Predictors of contact tracing completion and outcomes in tuberculosis: a 21-year retrospective cohort study. The international journal of tuberculosis and lung disease : the official journal of the International Union against Tuberculosis and Lung Disease. 2014;18:640-6.

6. Public Health England: LTBI testing and treatment programme for migrants: 2020 report.

7. Public Health England: Tuberculosis in England. 2020 report.

8. Public Health England: LTBI testing and treatment programme for migrants 2020: data tables.

9. Clifford S, Barber N, Horne R. Understanding different beliefs held by adherers, unintentional nonadherers, and intentional nonadherers: Application of the Necessity–Concerns Framework. Journal of psychosomatic research. 2008;64:41-6.

10. NICE. Medicines Adherence: Involving Patients in Decisions about Prescribed Medicines and Supporting Adherence: Full Guideline. Clinical Guideline CG76. 2009.

11. Horne R, Cooper V, Wileman V, et al. Supporting adherence to medicines for long-term conditions: A perceptions and practicalities approach based on an extended common-sense model. European Psychologist. 2019;24:82-96.

12. Liu X, Lewis JJ, Zhang H, et al. Effectiveness of Electronic Reminders to Improve Medication Adherence in Tuberculosis Patients: A Cluster-Randomised Trial. PLoS Med. 2015;12:e1001876.

For peer review only - http://bmjopen.bmj.com/site/about/guidelines.xhtml

13. Holzschuh EL, Province S, Johnson K, et al. Use of Video Directly Observed Therapy for Treatment of Latent Tuberculosis Infection - Johnson County, Kansas, 2015. MMWR Morbidity and mortality weekly report. 2017;66:387-9.

14. Lam CK, McGinnis Pilote K, Haque A, et al. Using Video Technology to Increase Treatment Completion for Patients With Latent Tuberculosis Infection on 3-Month Isoniazid and Rifapentine: An Implementation Study. Journal of medical Internet research. 2018;20:e287.

15. World Health Organization. Overcoming key barriers to scale up tuberculosis preventive treatment (TPT) A Call to Action. https://www.who.int/tb/CalltoactionTPT_scaleup.pdf?u=f093a7c38a3780cd9504f8d9d&id=135bdee6af&e=09bced52fa Accessed 18 May 2021.

16. Sterling TR, Villarino ME, Borisov AS, et al. Three months of rifapentine and isoniazid for latent tuberculosis infection. The New England journal of medicine. 2011;365:2155-66.

17. Zenner D, Beer N, Harris RJ, et al. Treatment of Latent Tuberculosis Infection: An Updated Network Meta-analysis. Ann Intern Med. 2017;167:248-55.

18. Swindells S, Ramchandani R, Gupta A, et al. One Month of Rifapentine plus Isoniazid to Prevent HIV-Related Tuberculosis. The New England journal of medicine. 2019;380:1001-11.

19. Horne R, Chapman SC, Parham R, et al. Understanding patients' adherence-related beliefs about medicines prescribed for long-term conditions: a meta-analytic review of the Necessity-Concerns Framework. PloS one. 2013;8:e80633.

20. Surey J, Stagg HR, Yates TA, et al. An open label, randomised controlled trial of rifapentine versus rifampicin based short course regimens for the treatment of latent tuberculosis in England: the HALT LTBI pilot study. BMC Infectious Diseases. 2021;21:90.

21. Public Health England: Latent TB Testing and Treatment for Migrants. A practical guide for commissioners and practitioners. 2015.

22. evriMED1000 Dispenser https://www.wisepill.com/wisepill-rm1000 Accessed 9 July 2021 [

23. Martinson NA, Barnes GL, Moulton LH, et al. New Regimens to Prevent Tuberculosis in Adults with HIV Infection. New England Journal of Medicine. 2011;365:11-20.

For peer review only - http://bmjopen.bmj.com/site/about/guidelines.xhtml

24. Sterling TR, Villarino ME, Borisov AS, et al. Three Months of Rifapentine and Isoniazid for Latent Tuberculosis Infection. New England Journal of Medicine. 2011;365:2155-66.

25. Office for National Statistics. Families and households in the UK: 2020. https://www.ons.gov.uk/peoplepopulationandcommunity/birthsdeathsandmarriages/families/bulletins/familiesandhouseholds/2020 accessed 15 Sep 2021.

26. Public Health England: Tuberculosis in England. 2019 report.

27. Howard DR, Brown JM, Todd S, et al. Recommendations on multiple testing adjustment in multi-arm trials with a shared control group. Stat Methods Med Res. 2018;27:1513-30.

28. Choodari-Oskooei B, Bratton DJ, Gannon MR, et al. Adding new experimental arms to randomised clinical trials: Impact on error rates. Clin Trials. 2020;17:273-84.

29. Wason JMS, Stecher L, Mander AP. Correcting for multiple-testing in multi-arm trials: is it necessary and is it done? Trials. 2014;15:364.

30. U.S. Department of Health and Human Services, National Institutes of Health, National Institute of Allergy and Infectious Diseases, Division of AIDS. Division of AIDS (DAIDS) Table for Grading the Severity of Adult and Pediatric Adverse Events, Corrected Version 2.1. [July 2017]. Available from: https://rsc.niaid.nih.gov/sites/default/files/daidsgradingcorrectedv21.pdf Accessed 11 September 2020.

For peer review only - http://bmjopen.bmj.com/site/about/guidelines.xhtml

580 **Box 1. Study inclusion and exclusion criteria**

**Inclusion criteria**
1. Aged ≥16 years to ≤65 at screening<br>2. LTBI diagnosis defined on the basis of all of the following:<br>(a) a positive result on an Interferon Gamma Release Assay (IGRA), Tuberculin Skin Test (TST) or C-Tb skin test and<br>(b) negative TB symptoms at screening and<br>(c) no signs of active TB on a Chest X-ray<br>3. Eligible for LTBI treatment at TB clinics and national LTBI screening services based on NICE guidelines, which means having one or more of the following :<br>• Recent infection (contact tracing);<br>• New entrants at risk (i.e., those that immigrated < 5 years from countries with a high incidence of TB, which is defined as ≥ 40 cases/100,000 population); or<br>• Individuals who are assessed in the TB clinic for latent TB testing, or have been referred for treatment following testing by specialities or departments within primary or secondary care settings<br>4. Agree to LTBI treatment<br>5. Willing and able to provide written informed consent
**Exclusion criteria**
1. Patients weighing <30 kg.<br>2. Need for medications that cannot be safely taken together with study drugs (e.g. protease inhibitors in people living with HIV and people with refractory epilepsy taking phenytoin/carbamazepine)<br>3. Any medical condition deserving priority of treatment (such as: porphyria, malabsorption syndromes, Clostridium difficile-Associated Diarrhoea and other conditions)<br>4. History of sensitivity/intolerance to isoniazid or rifamycins<br>5. Individuals with documented liver disease, defined as:<br>• LFT (ALT/AST/bilirubin) over three times upper limit of normal (ULN) at baseline. This reflects normal clinical practice. For participant safety, liver function tests are carried on a regular basis. One abnormal value prevents the patient from participating on the study.<br>• Clinical diagnosis of cirrhosis (jaundice, hematemesis, ascites or previous episodes of liver encephalopathy),<br>• HbsAg positive or HCV antibody positive and deemed ineligible for LTBI treatment by the clinician<br>6. Intending to move outside of the treatment locality within 20 weeks of starting treatment<br>7. Individuals who would usually be offered LTBI treatment under Directly Observed Therapy (DOT) as part of enhanced case management in complex cases such as those from under-served groups (such as people who are homeless, misuse substances, have been in prison or who are vulnerable migrants).<br>8. Use of another experimental investigational medicinal product that is likely to interfere with the study medication within 3 months of study enrolment.<br>9. Women who are breastfeeding, pregnant, or of childbearing potential who do not agree to use an effective method of contraception from the time consent is signed until 4 weeks after treatment discontinuation or completion. Males whose partners are of child-bearing potential must also agree to use an effective method of contraception.<br>10. Women of child bearing potential without a negative urine pregnancy test within 7 days prior to being registered for trial treatment.

581

For peer review only - http://bmjopen.bmj.com/site/about/guidelines.xhtml

Table 1. Doses of study treatment

	Body weight		
ARM 1 and 2: rifampicin plus isoniazid once daily for 90 doses (3 months)	< 50 KG	≥ 50 kg	
	3 x Isoniazid/Rifampicin fixed dose combination (150/100)	2 x Isoniazid/Rifampicin fixed dose combination (300/150)	
ARM 3 and 4: rifapentine plus isoniazid once weekly for 12 doses (3 months)	30 to < 32 KG	32 to < 50 kg	≥ 50 kg
	Rifapentine 600 mg + Isoniazid 15 mg/kg	Rifapentine 750 mg + Isoniazid 15 mg/kg	Rifapentine 900 mg + Isoniazid 15 mg/kg (900 mg maximum)
ARM 5 and 6: rifapentine plus isoniazid once daily for 28 doses (one month)	30 to < 35 kg	35 to ≤ 45 kg	≥ 45kg
	Rifapentine 300 mg + 300mg Isoniazid	Rifapentine 450 mg + 300 mg isoniazid	Rifapentine 600 mg + 300 mg isoniazid

For peer review only

Table 2. Definition of the estimands for the primary analyses

**Attribute**	**Definition**
Treatments	The primary analyses are based on the following comparisons:<br>a) Arm 3 vs Arm 1- ie 3HP + routine treatment support vs 3HR + routine treatment support<br>b) Arm 5 vs Arm 1- ie 1HP + routine treatment support vs 3HR + routine treatment support<br>c) Arm 2 vs Arm 1- ie 3HR + additional treatment support vs 3HR + routine treatment support<br>If comparison (a) shows 3HP improves adherence compared to 3HR, then additional treatment support will be formally tested for 3HP by comparing Arm 4 vs Arm 3 – ie 3HP + additional treatment support vs 3HP + routine treatment support. Additional treatment support will be similarly assessed for 1HP.
Population	Adults aged 16 to 65 years diagnosed with LTBI and eligible for LTBI treatment.
Endpoint	Adequate treatment adherence, defined as taking ≥90% of allocated doses within the allowable time-frame**.**
Intercurrent events	The main intercurrent events and how they will be handled in the estimand are as follows:<br>• Failure to collect all prescriptions- composite and treatment policy strategies lead to same estimated effect.<br>• Early treatment discontinuation for any reason including adverse event(s) and active TB: a treatment policy strategy will be used, ie the participant is considered to have stopped treatment regardless of the occurrence of the intercurrent event.
Population-level summary measure	Risk ratio for adequate treatment adherence comparing the relevant arms.

For peer review only

For peer review only - http://bmjopen.bmj.com/site/about/guidelines.xhtml

592 **Table 3. Definitions of adverse events and reactions**

Term	Definition
Adverse Event (AE)	Any untoward medical occurrence in a patient or clinical trial participant to whom a medicinal product has been administered including occurrences that are not necessarily caused by or related to that product.
Adverse Reaction (AR)	Any untoward and unintended response to an investigational medicinal product related to any dose administered.
Unexpected Adverse Reaction (UAR)	An adverse reaction, the nature or severity of which is not consistent with the information about the medicinal product in question set out in approved Reference Safety Information for that product in the trial.
Serious Adverse Event (SAE) or Serious Adverse Reaction (SAR) or Suspected Unexpected Serious Adverse Reaction (SUSAR)	Any adverse event, adverse reaction or unexpected adverse reaction that:<br>▪ Results in death<br>▪ Is life-threatening*<br>▪ Requires hospitalisation or prolongation of existing hospitalisation**<br>▪ Results in persistent or significant disability or incapacity<br>▪ Consists of a congenital anomaly or birth defect<br>▪ Is another important medical condition***

593 *The term life-threatening in the definition of a serious event refers to an event in which the patient is at risk of death at the
594 time of the event; it does not refer to an event that hypothetically might cause death if it were more severe, for example,
595 a silent myocardial infarction.
596
597 **Hospitalisation is defined as an inpatient admission, regardless of length of stay, even if the hospitalisation is a
598 precautionary measure for continued observation. Hospitalisations for a pre-existing condition, that has not worsened or
599 for an elective procedure do not constitute an SAE.
600
601 *** Medical judgement should be exercised in deciding whether an AE or AR is serious in other situations. The following
602 should also be considered serious: important AEs or ARs that are not immediately life-threatening or do not result in
603 death or hospitalisation but may jeopardise the subject or may require intervention to prevent one of the other
604 outcomes listed in the definition above; for example, a secondary malignancy, an allergic bronchospasm requiring
605 intensive emergency treatment, seizures or blood dyscrasias that do not result in hospitalisation or development of drug
606 dependency.

607

For peer review only

608 **Figures**

609 **Figure 1. Trial schema**

610

611

For peer review only

For peer review only - http://bmjopen.bmj.com/site/about/guidelines.xhtml

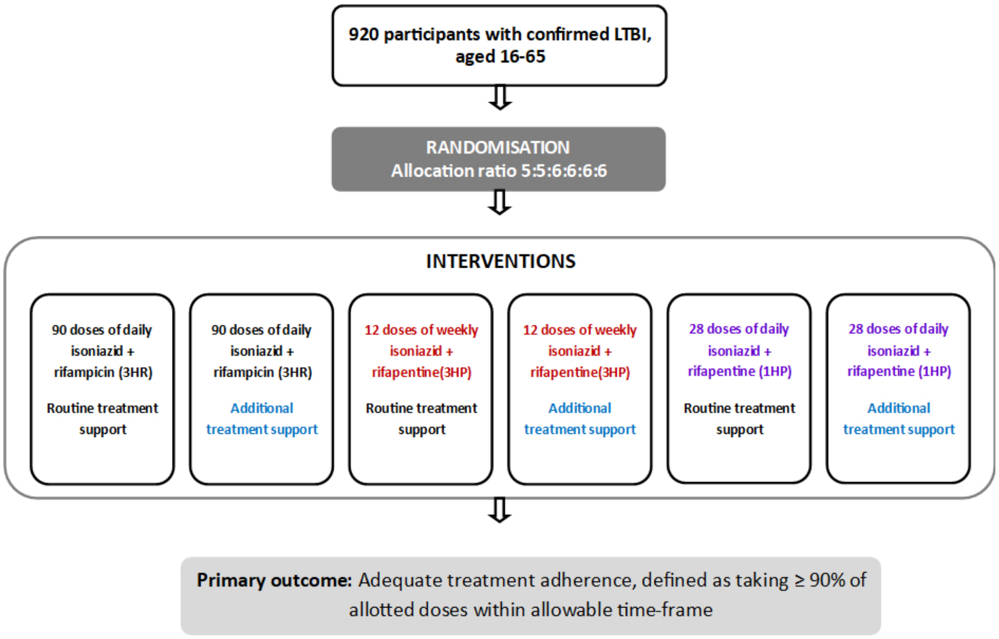

Figure 1. Trial schema

511x332mm (47 x 47 DPI)

STANDARD PROTOCOL ITEMS: RECOMMENDATIONS FOR INTERVENTIONAL TRIALS

SPIRIT 2013 Checklist: Recommended items to address in a clinical trial protocol and related documents*

Section/item	Item No	Description	Addressed on page number
**Administrative information**			
Title	1	Descriptive title identifying the study design, population, interventions, and, if applicable, trial acronym	1
Trial registration	2a	Trial identifier and registry name. If not yet registered, name of intended registry	3
	2b	All items from the World Health Organization Trial Registration Data Set	In the original protocol
Protocol version	3	Date and version identifier	In the original protocol
Funding	4	Sources and types of financial, material, and other support	21
Roles and responsibilities	5a	Names, affiliations, and roles of protocol contributors	1, 21
	5b	Name and contact information for the trial sponsor	1
	5c	Role of study sponsor and funders, if any, in study design; collection, management, analysis, and interpretation of data; writing of the report; and the decision to submit the report for publication, including whether they will have ultimate authority over any of these activities	21
	5d	Composition, roles, and responsibilities of the coordinating centre, steering committee, endpoint adjudication committee, data management team, and other individuals or groups overseeing the trial, if applicable (see Item 21a for data monitoring committee)	In the original protocol

For peer review only

**Introduction**			
Background and rationale	6a	Description of research question and justification for undertaking the trial, including summary of relevant studies (published and unpublished) examining benefits and harms for each intervention	6-8
	6b	Explanation for choice of comparators	6-8
Objectives	7	Specific objectives or hypotheses	8-9
Trial design	8	Description of trial design including type of trial (eg, parallel group, crossover, factorial, single group), allocation ratio, and framework (eg, superiority, equivalence, noninferiority, exploratory)	9-10
**Methods: Participants, interventions, and outcomes**			
Study setting	9	Description of study settings (eg, community clinic, academic hospital) and list of countries where data will be collected. Reference to where list of study sites can be obtained	10
Eligibility criteria	10	Inclusion and exclusion criteria for participants. If applicable, eligibility criteria for study centres and individuals who will perform the interventions (eg, surgeons, psychotherapists)	10
Interventions	11a	Interventions for each group with sufficient detail to allow replication, including how and when they will be administered	10-12
	11b	Criteria for discontinuing or modifying allocated interventions for a given trial participant (eg, drug dose change in response to harms, participant request, or improving/worsening disease)	In the original protocol
	11c	Strategies to improve adherence to intervention protocols, and any procedures for monitoring adherence (eg, drug tablet return, laboratory tests)	11-12
	11d	Relevant concomitant care and interventions that are permitted or prohibited during the trial	In the original protocol
Outcomes	12	Primary, secondary, and other outcomes, including the specific measurement variable (eg, systolic blood pressure), analysis metric (eg, change from baseline, final value, time to event), method of aggregation (eg, median, proportion), and time point for each outcome. Explanation of the clinical relevance of chosen efficacy and harm outcomes is strongly recommended	13-14
Participant timeline	13	Time schedule of enrolment, interventions (including any run-ins and washouts), assessments, and visits for participants. A schematic diagram is highly recommended (see Figure)	12-13, Figure 1

For peer review only - http://bmjopen.bmj.com/site/about/guidelines.xhtml

Sample size	14	Estimated number of participants needed to achieve study objectives and how it was determined, including clinical and statistical assumptions supporting any sample size calculations	14
Recruitment	15	Strategies for achieving adequate participant enrolment to reach target sample size	10
**Methods: Assignment of interventions (for controlled trials)**			
Allocation:			
Sequence generation	16a	Method of generating the allocation sequence (eg, computer-generated random numbers), and list of any factors for stratification. To reduce predictability of a random sequence, details of any planned restriction (eg, blocking) should be provided in a separate document that is unavailable to those who enrol participants or assign interventions	15
Allocation concealment mechanism	16b	Mechanism of implementing the allocation sequence (eg, central telephone; sequentially numbered, opaque, sealed envelopes), describing any steps to conceal the sequence until interventions are assigned	15
Implementation	16c	Who will generate the allocation sequence, who will enrol participants, and who will assign participants to interventions	15
Blinding (masking)	17a	Who will be blinded after assignment to interventions (eg, trial participants, care providers, outcome assessors, data analysts), and how	NA
	17b	If blinded, circumstances under which unblinding is permissible, and procedure for revealing a participant's allocated intervention during the trial	NA
**Methods: Data collection, management, and analysis**			
Data collection methods	18a	Plans for assessment and collection of outcome, baseline, and other trial data, including any related processes to promote data quality (eg, duplicate measurements, training of assessors) and a description of study instruments (eg, questionnaires, laboratory tests) along with their reliability and validity, if known. Reference to where data collection forms can be found, if not in the protocol	15-16
	18b	Plans to promote participant retention and complete follow-up, including list of any outcome data to be collected for participants who discontinue or deviate from intervention protocols	15-16

Data management	19	Plans for data entry, coding, security, and storage, including any related processes to promote data quality (eg, double data entry; range checks for data values). Reference to where details of data management procedures can be found, if not in the protocol	15-16
Statistical methods	20a	Statistical methods for analysing primary and secondary outcomes. Reference to where other details of the statistical analysis plan can be found, if not in the protocol	16-17
	20b	Methods for any additional analyses (eg, subgroup and adjusted analyses)	16-17
	20c	Definition of analysis population relating to protocol non-adherence (eg, as randomised analysis), and any statistical methods to handle missing data (eg, multiple imputation)	16-17
**Methods: Monitoring**			
Data monitoring	21a	Composition of data monitoring committee (DMC); summary of its role and reporting structure; statement of whether it is independent from the sponsor and competing interests; and reference to where further details about its charter can be found, if not in the protocol. Alternatively, an explanation of why a DMC is not needed	18
	21b	Description of any interim analyses and stopping guidelines, including who will have access to these interim results and make the final decision to terminate the trial	18
Harms	22	Plans for collecting, assessing, reporting, and managing solicited and spontaneously reported adverse events and other unintended effects of trial interventions or trial conduct	17
Auditing	23	Frequency and procedures for auditing trial conduct, if any, and whether the process will be independent from investigators and the sponsor	18
**Ethics and dissemination**			
Research ethics approval	24	Plans for seeking research ethics committee/institutional review board (REC/IRB) approval	20
Protocol amendments	25	Plans for communicating important protocol modifications (eg, changes to eligibility criteria, outcomes, analyses) to relevant parties (eg, investigators, REC/IRBs, trial participants, trial registries, journals, regulators)	20

For peer review only - http://bmjopen.bmj.com/site/about/guidelines.xhtml

Consent or assent	26a	Who will obtain informed consent or assent from potential trial participants or authorised surrogates, and how (see Item 32)	20
	26b	Additional consent provisions for collection and use of participant data and biological specimens in ancillary studies, if applicable	In the original protocol
Confidentiality	27	How personal information about potential and enrolled participants will be collected, shared, and maintained in order to protect confidentiality before, during, and after the trial	In the original protocol
Declaration of interests	28	Financial and other competing interests for principal investigators for the overall trial and each study site	21
Access to data	29	Statement of who will have access to the final trial dataset, and disclosure of contractual agreements that limit such access for investigators	20
Ancillary and post-trial care	30	Provisions, if any, for ancillary and post-trial care, and for compensation to those who suffer harm from trial participation	In the original protocol
Dissemination policy	31a	Plans for investigators and sponsor to communicate trial results to participants, healthcare professionals, the public, and other relevant groups (eg, via publication, reporting in results databases, or other data sharing arrangements), including any publication restrictions	20
	31b	Authorship eligibility guidelines and any intended use of professional writers	In the original protocol
	31c	Plans, if any, for granting public access to the full protocol, participant-level dataset, and statistical code	20
**Appendices**			
Informed consent materials	32	Model consent form and other related documentation given to participants and authorised surrogates	Submitted to HRA and app
Biological specimens	33	Plans for collection, laboratory evaluation, and storage of biological specimens for genetic or molecular analysis in the current trial and for future use in ancillary studies, if applicable	NA

*It is strongly recommended that this checklist be read in conjunction with the SPIRIT 2013 Explanation & Elaboration for important clarification on the items. Amendments to the protocol should be tracked and dated. The SPIRIT checklist is copyrighted by the SPIRIT Group under the Creative Commons "Attribution-NonCommercial-NoDerivs 3.0 Unported" license.

For peer review only - http://bmjopen.bmj.com/site/about/guidelines.xhtml

BMJ Open

Evaluating the effect of short-course rifapentine-based regimens with or without enhanced behaviour-targeted treatment support on adherence and completion of treatment for latent tuberculosis infection among adults in the UK (RID-TB: Treat): protocol for an open-label, multicentre, randomised controlled trial

Journal:	*BMJ Open*
Manuscript ID	bmjopen-2021-057717.R1
Article Type:	Protocol
Date Submitted by the Author:	18-Jan-2022

Complete List of Authors:	Rangaka , Molebogeng ; University College London; University of Cape Town<br>Hamada, Yohhei; University College London, Institute for Global Health<br>Duong, Trinh; MRC Clinical Trials Unit at UCL<br>Bern, Henry; MRC Clinical Trials Unit at UCL<br>Calvert, Joanna ; MRC Clinical Trials Unit at UCL<br>Francis, Marie; University College London<br>Clarke, Amy; University College London, School of Pharmacy<br>Ghanouni, Alex; UCL School of Pharmacy<br>Layton, Charlotte; MRC Clinical Trials Unit at UCL<br>Hack, Vanessa ; University College London, Institute for Global Health<br>Owen-Powell, Ellen; MRC Clinical Trials Unit at UCL<br>Surey, Julian; University College London<br>Sanders, Karen; MRC Clinical Trials Unit at UCL<br>Booth, Helen; University College London Hospitals NHS Foundation Trust<br>Crook, Angela; University College London Institute of Clinical Trials and Methodology, MRC Clinical Trials Unit<br>Griffiths, Chris; Barts and The London School of Medicine and Dentistry<br>Horne, Robert; UCL School of Pharmacy<br>Kunst, Heinke; Queen Mary University of London, Department of Respiratory Medicine<br>Lipman, Marc; Royal Free London Hospital NHS Foundation Trust; University College London<br>Mandelbaum, Mike; TB Alert<br>White, Peter; Imperial College, School of Public Health; Public Health England, Modelling and Economics Unit<br>Zenner, Dominik; University College London Institute for Global Health; Queen Mary University of London, Wolfson Institute for Population Health Barts and the London School of Medicine and Dentistry<br>Abubakar, Ibrahim; University College London

**Primary Subject Heading**:	Infectious diseases
Secondary Subject Heading:	Respiratory medicine

Keywords:	Tuberculosis < INFECTIOUS DISEASES, Clinical trials < THERAPEUTICS, PREVENTIVE MEDICINE

SCHOLARONE™
Manuscripts

Evaluating the effect of short-course rifapentine-based regimens with or without enhanced behaviour-targeted treatment support on adherence and completion of treatment for latent tuberculosis infection among adults in the UK (RID-TB: Treat): protocol for an open-label, multicentre, randomised controlled trial

Molebogeng X. Rangaka[1,2], Yohhei Hamada[1], Trinh Duong[3], Henry Bern[3], Joanna Calvert[3], Marie Francis[1], Amy Louise Clarke[4], Alex Ghanouni[4], Charlotte Layton[3], Vanessa Hack[1], Ellen Owen-Powell[3], Julian Surey[1], Karen Sanders[3], Helen L. Booth[5], Angela M. Crook[3], Chris Griffiths[6], Rob Horne[4], Heinke Kunst[7], Marc Lipman[8,9], Mike Mandelbaum[10], Peter J. White[11, 12], Dominik Zenner[1],[6], Ibrahim Abubakar[1]

[1] Institute for Global Health, University College London, London, UK

[2] University of Cape Town, Cape Town, South Africa

[3] MRC Clinical Trials Unit at UCL, Institute of Clinical Trials and Methodology, London, UK

[4] Centre for Behavioural Medicine, UCL School of Pharmacy, London, UK

[5] North Central London Tuberculosis Service, Whittington Health NHS Trust and University College London Hospitals NHS Foundation Trust, UK London, UK

[6] Wolfson Institute for Population Health Barts and the London School of Medicine and Dentistry, Queen Mary University of London, UK

[7] Blizard Institute, Barts and The London School of Medicine and Dentistry, Queen Mary University of London, UK

[8] Royal Free London Hospital NHS Foundation Trust, London, UK

[9] UCL Respiratory, Division of Medicine, University College London, London, UK.

[10] TB Alert, London, UK

[11] MRC Centre for Global Infectious Disease Analysis, Imperial College London, London, UK

[12] Modelling and Economics Unit, National Infection Service, Public Health England, London, UK

Corresponding author

Molebogeng X Rangaka

Institute for Global Health, University College

30 Guilford Street, London WC1N 1EH

For peer review only

Email: l.rangaka@ucl.ac.uk

Phone: +44-7552-526872

Keywords: clinical trial; LTBI; medication adherence; rifapentine; tuberculosis

For peer review only

Abstract

Introduction

The successful scale-up of a latent tuberculosis infection (LTBI) testing and treatment programme is essential to achieve TB elimination. However, poor adherence compromises its therapeutic effectiveness. Novel rifapentine-based regimens and treatment support based on behavioural science theory may improve treatment adherence and completion.

Methods and analysis

A pragmatic multi-centre, open-label, randomised controlled trial assessing the effect of novel short-course rifapentine-based regimens for TB prevention and additional theory-based treatment support on treatment adherence against standard-of-care. Participants aged between 16 and 65 who are eligible to start TB preventive therapy will be recruited in England. 920 participants will be randomized to one of six arms with allocation ratio of 5:5:6:6:6:6: (1) daily isoniazid + rifampicin for three months (3HR), routine treatment support (control); (2) 3HR, additional treatment support; (3) weekly isoniazid + rifapentine for three months (3HP), routine treatment support; (4) weekly 3HP, additional treatment support; (5) daily isoniazid + rifapentine for one month (1HP), routine treatment support; (6) daily 1HP, additional treatment support. Additional treatment support comprises reminders using an electronic pillbox, a short animation, and leaflets based on the Perceptions and Practicalities Approach. The primary outcome is adequate treatment adherence, defined as taking ≥90% of allocated doses within the pre-specified treatment period, measured by electronic pillboxes. Secondary outcomes include safety and TB incidence within 12 months. We will conduct process evaluation of the trial interventions and assess intervention acceptability and fidelity and mechanisms for effect and estimate the cost-effectiveness of novel regimens. The protocol was developed with Patient and Public Involvement, which will continue throughout the trial.

Ethics and dissemination

For peer review only - http://bmjopen.bmj.com/site/about/guidelines.xhtml

Ethics approval has been obtained from The NHS Health Research Authority (20/LO/1097). All participants will be required to provide written informed consent. We will share the results in peer-reviewed journals.

Trial registration number EudraCT 2020-004444-29

For peer review only - http://bmjopen.bmj.com/site/about/guidelines.xhtml

Article summary

Strengths and limitations of this study

- The trial allows evaluation of both the effect of two rifapentine-based regimens compared to the standard 3-month daily rifampicin plus isoniazid, and the effect of additional treatment support compared to routine support, on LTBI treatment adherence.
- We will perform process evaluation of the trial interventions, including assessment of intervention acceptability and fidelity, and economic evaluation, which will provide additional evidence to inform treatment options and treatment support.
- The trial is powered to evaluate novel rifapentine-based regimens compared to the standard daily rifampicin plus isoniazid (3HR) and the effect of additional treatment support compared to routine support; however, it does not have sufficient power to evaluate all possible comparisons such as 3-month weekly rifapentine plus isoniazid vs 1-month daily rifapentine plus isoniazid.
- The trial will be conducted in England largely in migrant populations eligible for the LTBI screening programme and contacts of TB patients and thus limiting generalisability to these populations and similar settings.
- Adherence will be measured using electronic pillboxes in all arms while reminders will be activated only in arms with additional treatment support; however, this may impact adherence in control groups.

For peer review only - http://bmjopen.bmj.com/site/about/guidelines.xhtml

INTRODUCTION

Successful implementation of screening and treatment for latent tuberculosis infection (LTBI) is critical to further reduce TB incidence globally and achieve TB elimination in low TB incidence countries.(1) A recent call to action issued by the World Health Organization urged for accelerating the scale-up of treatment of LTBI, particularly to mitigate the negative impact from the disruption of TB services caused by the pandemic of COVID-19.(2)

Tuberculosis (TB) in England disproportionately affects underserved communities, such as migrants and homeless people, who consequently experience higher disease burden and worse clinical outcomes. Consequently, in England, LTBI screening and treatment for high risk groups such as new migrants from high TB incidence countries is recognized as an essential strategy to achieve TB elimination.(3) Contact tracing, including testing and treatment of LTBI among contacts, is another essential component of the TB strategy for England.(3)

Achieving optimal treatment adherence and completion is essential to ensure the efficacy of treatment for LTBI and to achieve commensurate reductions in TB incidence. Standard therapeutic options in the UK include 3-months of self-administered daily isoniazid/rifampicin and 6-months of daily isoniazid; the former regimen is often prescribed because of the availability of fixed-dose formulations and its shorter duration. Whilst these regimen are efficacious in preventing disease, their effectiveness is limited by low treatment adherence and completion rates.(4, 5) According to data from England in migrants whose treatment outcome is known, 75% completed LTBI treatment between 2019 and 2020.(6, 7) The proportion of people who completed treatment varied by Clinical Commission Group (CCG), which was less than 70% in several CCGs.(8)

People with LTBI may need additional support to adhere to effective treatments. Treatment non-adherence can be intentional or unintentional, and is driven by a person's motivation and ability to take medicine as prescribed, respectively.(9) Motivation is influenced by our perceptions (e.g. beliefs and preferences) and ability is determined by practical factors (e.g. internal capacity and resource).(9)

For peer review only - http://bmjopen.bmj.com/site/about/guidelines.xhtml

These principles are operationalised as part of the Perceptions and Practicalities approach to supporting adherence (PAPA) and are applied in NICE guidelines.(10) The Necessity and Concerns Framework (NCF) further explains how patients' motivation to engage with treatment is based on their perceived necessity for, and concerns about the treatment.(11) Necessity beliefs are influenced by perceptions of the health threat (e.g LTBI) and interpretation of symptoms. The asymptomatic nature of LTBI may negatively impact necessity beliefs, and heighten treatment concerns. As such, intervention to support treatment adherence in people with LTBI will likely be more effective if they address patient beliefs and concerns around treatment, in addition to removing practical barriers.

The need to understand perceptual and practical barriers to treatment adherence, and the potential of advancing technology and drug regimens in the NHS has been highlighted. Some mobile/digital technology (mHealth) has been shown to improve adherence in TB disease studies. A recent study in China found electronic reminders, using specially designed electronic medication monitors, improved treatment adherence in such TB patients, but multiple two-way daily text messaging reminders, didactic in nature, did not.(12) Most of the evidence available is on TB disease with little research on mHealth interventions to improve LTBI treatment adherence.(13, 14) Another call to action issued by the World Health Organization suggested TB preventive treatment programmes should consider communication technologies for medication adherence support.(15) The evidence on mHealth interventions for LTBI treatment would contribute to their global scale-up.

Another approach to promote better treatment adherence and completion is to decrease the complexity of current LTBI regimens. A regimen that is given once weekly may result in better treatment completion than the current daily 3-month regimen. A randomised controlled trial demonstrated that a new regimen of 12 doses of weekly rifapentine and isoniazid (3HP) delivered through direct observation (i.e. with patients being supervised taking each dose) is non-inferior to 9 months of daily isoniazid.(16) Our network meta-analysis suggests that 3HP has similar efficacy to the UK standard-of-care of a 12-week, daily isoniazid/rifampicin regimen (3HR).(17) Furthermore, a recent trial in people living with HIV (23% with LTBI as demonstrated by a positive TST and/or IGRA result)

For peer review only - http://bmjopen.bmj.com/site/about/guidelines.xhtml

demonstrated non-inferiority of daily one-month rifapentine plus isoniazid (1HP) compared to 9 months of daily isoniazid.(18) The one-month regimen resulted in better adherence and fewer serious adverse events. Based on this study, and by extrapolating to HIV-negative individuals, newly published WHO guidelines recommend this regimen regardless of HIV status. However, there is no published evaluation of whether these more expensive rifapentine-based regimens lead to better treatment completion than the current daily administered UK standard-of-care. In particular, evidence is limited on the use of 3HP with patient self-administration and no study has compared its completion with 3HR.

To develop tools to reduce TB rates, we need to evaluate advancing technology and drug regimens, but also understand the barriers and enablers of adherence.(3) To date, adherence interventions have predominantly focused on removing practical barriers to adherence (e.g reminder of shortening the drug regimen). However, such approaches applied in isolation ignore patient beliefs. LTBI is asymptomatic which means patients might have a disconnect between medical advice and their perceived need for treatment.(19) NICE guidelines recommend a Perceptions and Practicalities Approach (PAPA) to adherence support, whereby beliefs (necessity and concerns) are elicited and addressed in addition to practical barriers.(10, 11)

We previously conducted the HALT-LTBI study, a pilot study assessing the safety and treatment completion of 3HP compared to standard care.(20) HALT-LTBI demonstrated the feasibility of recruiting LTBI patients to such a trial; no serious adverse events defined as grade 3 or more were reported, supporting the safety of rifapentine and isoniazid regimens in individuals eligible for LTBI treatment in the UK. 78% and 68% of participants completed treatment in the experimental and standard-of-care arms, respectively, but the pilot was not powered to detect differences in treatment completion. Thus, we will conduct a fully powered trial to compare treatment adherence and adverse events of novel 3HP and 1HP regimens compared with 3HR and to assess the effect of additional treatment support in participants given each regimen.

Objectives

For peer review only

For peer review only - http://bmjopen.bmj.com/site/about/guidelines.xhtml

The primary objective of this trial is to assess the effect of novel rifapentine-based regimens (3HP or 1HP) compared to the standard 90-dose daily rifampicin plus isoniazid (3HR), and the effect of additional treatment support compared to routine support, on LTBI treatment adherence.

The secondary objectives are: 1) to evaluate the effect of LTBI treatment and additional treatment support using alternate measures of adherence outcome; and 2) to compare the frequency of adverse events whilst on treatment for LTBI, and development of TB within 12 months following treatment. Additionally, we will evaluate the process of delivering the adherence intervention and examine intervention fidelity and acceptability as well as the cost-effectiveness of different treatment options and/or additional treatment support.

METHOD AND ANALYSIS

Trial design

A multi-centre open-label randomised controlled trial with the following six parallel groups (Figure 1):

ARM 1- Daily isoniazid + rifampicin for three months (3HR), routine treatment support (SOC; control arm)

ARM 2- Daily 3HR, additional treatment support

ARM 3- Weekly isoniazid + rifapentine for three months (3HP), routine treatment support

ARM 4- Weekly 3HP, additional treatment support

ARM 5- Daily isoniazid + rifapentine for one month (1HP), routine treatment support

ARM 6- Daily 1HP, additional treatment support

A factorial design was not chosen for several reasons. Firstly, it is anticipated that there will be an interaction between type of regimen and treatment support; additional treatment support is likely to confer a smaller benefit with 3HP/1HP compared to 3HR. Secondly, the power to detect the effect of an intervention would be reduced if the effect of the second intervention is greater than expected.

For peer review only - http://bmjopen.bmj.com/site/about/guidelines.xhtml

Study setting

The trial will recruit from secondary care sites that provide LTBI treatment in England, UK. RID-TB: Treat is part of a 5-year programme of work (RID-TB) which is funded by the National Institute for Health Research (NIHR) (RP-PG-0217-20009 https://dev.fundingawards.nihr.ac.uk/award/RP-PG-0217-20009). We expect to recruit participants from 15 care sites.

Study population

The trial will enrol populations who are eligible for treatment for LTBI according to the national guidance. We envisage that the majority of individuals eligible for this are contacts of persons diagnosed with TB disease, and/or migrants eligible for the national LTBI screening programme.(21) The LTBI migrant screening programme includes migrants who are aged 16 to 35 years, entered the UK from a high incidence country (≥150/100,000) or Sub-Saharan Africa within the last five years and had been previously living in that high incidence country for six months or longer.(21) Inclusion and exclusion criteria are shown in Box 1.

Participants will be identified from secondary care settings in the UK where persons eligible for treatment for LTBI are managed. Participants will be recruited individually, but if any participants share a household, they will be allocated to the same arm as the first person recruited from that household (effectively resulting in randomisation by household).

Non-English speakers will not be excluded from the trial. We will translate patient-facing materials and use interpreters to support non-English speaking participants.

Treatment

Participants who are randomized to arms 1 and 2 will receive the standard of care regimen: rifampicin plus isoniazid once daily for 90 doses (3HR).

For peer review only - http://bmjopen.bmj.com/site/about/guidelines.xhtml

Participants who are randomized to arms 3 and 4 will receive rifapentine plus isoniazid once weekly for 12 doses (3HP) and those who are randomized to arms 5 and 6 will receive rifapentine plus isoniazid once daily for 28 doses (1HP). Participants will be given a 1-month supply of the medications at every visit in general but it also depends on local practice as this is a pragmatic trial.

In order to account for missed doses and interruption of treatment due to adverse events, participants given 3HR or 3HP will have 16 weeks and those given 1HP will have 6 weeks to complete treatment. In the study by Swindells et al, participants were given 8 weeks to complete 1HP.(18) We have chosen 6 weeks to make the period proportionally similar to that for 3HR and 3HP. Clinicians will assess the need for treatment extension based on the assessment of adherence and review of reasons for non-adherence but should not extend beyond recommended grace periods.

In all arms, participants will receive vitamin B6 (pyridoxine). The dosages of study drugs are shown in Table 1.

Rifapentine and rifampicin are known to induce the hepatic cytochrome CYP450 enzyme system. Caution is recommended in using medications that are metabolized by this system. Concurrent use of protease inhibitors, hepatitis-C antiviral drugs, or praziquantel is not permitted.

Treatment support

Routine treatment support

Participants allocated to arms 1, 3, and 5 will receive routine treatment support. Participants will be given information about treatment for LTBI including expected adverse events and the importance of adherence, according to local practice. Adherence will be reviewed at each follow-up visit or remote consultation via self-reporting and/or pill count and discussed with the participant. An electronic pill monitor box, Wisepill EvriMed1000 (Wisepill, Somerset West, South Africa)(22) will collect the date

For peer review only - http://bmjopen.bmj.com/site/about/guidelines.xhtml

and time of each opening to collect information on adherence. However, it will be set to silent mode and not be used as an adherence reminder tool.

Intervention

Participants assigned to arms 2, 4, and 6 will receive a PAPA-based intervention designed to provide additional treatment support (i.e. in addition to routine treatment support).(11) Specifically, the intervention will consist of an animation which will 1) provide a rationale for treatment necessity and help people understand how LTBI treatment can help them to achieve a health goal that is important to them, 2) address common concerns about LTBI treatment and 3) address practical barriers to treatment (e.g. anchoring treatment to daily activities). The animation will be supported by a leaflet that covers misperceptions about LTBI testing and treatment, and other frequently asked questions. Participants will also be asked to set reminders using an electronic pill monitor box (Wisepill EvriMed). The electronic pillbox allows two modes of reminders: audio alarm from the box or text-message to participants' mobile phones.

The reminder can be set at pre-specified times and can also be activated to send a reminder when the pill box is not opened. Site staff will discuss options with each participant and set reminders according to their preferences. Participants can opt not to receive reminders before or at the time of intended medication intake. However, they will still be reminded when the box is not opened within a pre-specified time in a day and they will receive a supportive text message automatically sent by the pillbox. The mode of reminder can be further adjusted during the course of treatment as necessary upon discussion with a clinician. The pillbox will electronically collect the date and time of each opening.

Study assessment and follow-up

Screening, randomisation and baseline assessment

Randomisation and baseline assessment will occur on the same day (Week 0). In some cases, this may also be the same day as Screening. Following informed consent procedures, participants will be

For peer review only - http://bmjopen.bmj.com/site/about/guidelines.xhtml

screened for eligibility. A TB symptom screen and urine pregnancy test will be carried out, and data on the participant's TB risk group category will be collected. Demographic and medical history information will be collected. We will check the results of clinical, laboratory, and radiological assessments performed under routine care before entry to the trial to confirm eligibility. A TB symptom screen and urine pregnancy test will be repeated at the randomization/baseline visits unless the screening and randomisation visits occur on the same day.

Assessment of Adherence

Assessment of adherence will be primarily measured using the Wisepill, which collects the date and time of each opening. Adherence will also be measured through self-reporting and pill count under routine care either at physical clinic visits or remote consultations as per the local standard. Attending clinicians will count the number of remaining tablets. The difference between the number of tablets dispensed and the number returned will be calculated.

Clinical assessment during follow-up

As per usual practice, liver function tests (hepatic transaminases, ALT/AST, and total bilirubin) will be performed at week 2 for all participants. Afterwards, liver function tests will be performed at weeks 4, 8, 12, and 16 while on treatment and at completion, or at other times if deemed necessary by attending clinicians (e.g. abnormality in preceding tests, new onset of symptoms suggesting potential liver toxicity). These tests should be performed at any time during the treatment and post-treatment phase if the participant exhibits symptoms or signs of drug-induced liver injury (DILI).

Adverse events expected with study drugs will be clinically assessed at every visit. These include anorexia, nausea, vomiting, fatigue, weakness, jaundice, rash, peripheral neuropathy, and bruising.. Participants who already completed treatment and have no scheduled visits will be given a phone call at week 8, 12, 16, and 20 to check adverse events and TB signs and symptoms since the last dose.

At every physical visit or remote consultation, symptoms and signs of TB disease will be reviewed as well as concomitant medications using a brief questionnaire. There will be no formal study visits after completion of treatment.

For peer review only - http://bmjopen.bmj.com/site/about/guidelines.xhtml

For peer review only

Protocol treatment discontinuation

An individual participant may stop treatment early or trial participation be stopped early for any of the following reasons: Unacceptable toxicity or adverse event including (e.g. serious adverse events leading to discontinuation of treatment); intercurrent illness that prevents further treatment; active TB disease; any change in the participant's condition that justifies the discontinuation of treatment in the clinician's opinion; pregnancy; inadequate compliance with the protocol treatment that preclude treatment within allowable time-frame in the judgement of the treating physician; and withdrawal of consent for treatment by the participant.

For peer review only

Outcomes

Primary Outcome

The primary outcome is adequate treatment adherence, defined as taking ≥90% of allocated doses within the allowable time-frame from randomisation (binary outcome). For the primary analyses, treatment adherence is measured using an electronic monitor box

Secondary Outcomes

The secondary outcome measures are:

- Effectiveness: (1) Proportion of allocated doses missed over the treatment period (measured using monitor box); (2) Proportion of allocated pills missed over the treatment period (measured using pill counts); (3) Taking at least 90% of doses and pills over the treatment period (binary outcome assessed using both monitor box and pill counts); (4) Early study treatment discontinuation for any reason

- Safety: (1) Permanently stop study treatment due to drug-related adverse events (i.e. adverse reactions) ; (2) Experience Grade ≥3 adverse events; (3) Develop TB disease within 12 months.

Sample size

The six-arm design allows evaluation of:

- the effect of the novel treatment regimens (3HP and 1HP) versus standard-of-care regimen (3HR), under routine treatment support
- the effect of additional treatment support vs routine treatment support for each individual regimen

A total of 920 participants are to be recruited. This provides 80% power for each of the following comparisons:

- Arm 3 vs Arm 1- ie 3HP + routine treatment support vs 3HR + routine treatment support
- Arm 5 vs Arm 1- ie 1HP + routine treatment support vs 3HR + routine treatment support
- Arm 2 vs Arm 1- ie 3HR + additional treatment support vs 3HR + routine treatment support
- Arm 4 vs Arm 3- ie 3HP + additional treatment support vs 3HP + routine treatment support
- Arm 6 vs Arm 5- ie 1HP + additional treatment support vs 1HP + routine treatment support

The power calculations assume the following:

- 70% adherence rate in Arm 1
- 3HP and 1HP improve adherence rate by 15% (absolute difference) compared to 3HR, respectively, with routine treatment support(18, 23, 24)
- Compared to routine treatment support, additional treatment support improves adherence rate by 15% for 3HR, and 10% for 3HP and 1HP, respectively(12)
- 2-sided alpha 5% (see below for type I error considerations)
- average number of participants enrolled per household is 2, taking into account the average household size in UK.(25)

For peer review only - http://bmjopen.bmj.com/site/about/guidelines.xhtml

- intra-class correlation (ICC) within a household is 0.1

The 70% adherence rate assumed for Arm 1 is based on the 77% LTBI treatment completion rate reported from the Public Health England LTBI testing and treatment database for 2018.(26)

Randomisation and allocation

Participants will be randomised centrally using a computerised algorithm developed and maintained by the Medical Research Council Clinical Trials Unit at University College London (MRC-CTU). To randomise a participant, the information contained on a completed Randomisation Form will be entered into the secure online trial database by trial team members at the site who have been trained and authorised to randomise by the MRC-CTU. The database will automatically check for eligibility. Only those who meet all eligibility criteria will be able to be randomised. Randomisation will be performed using minimisation with an additional random element, to be balanced with respect to centre and TB exposure risk group.

Blinding

This is an open-label trial. Blinding of participants and care providers to the allocation group is not relevant since the primary objective of this trial is to examine the effect of shorter or weekly regimens and additional treatment support on treatment adherence.

Data collection methods and management

Adherence data will be collected through the Wisepill monitor box. Demographic and clinical information will be collected through clinical consultation and recorded on relevant worksheets. Development of TB within 12 months after starting treatment and outcomes of pregnancy that are found after enrolment will be collected using records held by NHS Digital, Public Health England, and/or the National TB register.

The trial will be conducted in compliance with the UK Data Protection Act 2018 (DPA number: Z6364106) and the EU Regulation General Data Protection Regulations 2016/679/ EC (GDPR) for protection of personal data.

For peer review only - http://bmjopen.bmj.com/site/about/guidelines.xhtml

Statistical methods

The estimands for the primary analyses are defined in Table 2. The primary analyses will compare the proportion of participants with adequate adherence between arms using the following approach:

a) Arm 3 vs Arm 1- ie 3HP + routine treatment support vs 3HR + routine treatment support
b) Arm 5 vs Arm 1- ie 1HP + routine treatment support vs 3HR + routine treatment support
c) Arm 2 vs Arm 1- ie 3HR + additional treatment support vs 3HR + routine treatment support

If comparison (a) shows 3HP improves adherence compared to 3HR, then additional treatment support will be formally tested for 3HP by comparing Arm 4 vs Arm 3 – ie 3HP + additional treatment support vs 3HP + routine treatment support; otherwise, the adherence rates will be compared between these arms as exploratory analyses. Additional treatment support will be similarly assessed for 1HP.

All randomised patients will be included in the primary analyses, apart from those subsequently found to have had TB disease at baseline but enrolled in error (modified intention-to-treat approach). The risk ratio (with 95% confidence interval) for adequate treatment adherence comparing the relevant arms will be estimated using log-binomial generalised linear mixed models (GLMMs), allowing for intra-household correlation.

Type I error adjustment for multiple comparisons is not deemed necessary since:

- The research hypotheses corresponding to comparisons (a), (b) and (c) are considered sufficiently distinct.(27-29)
- The effect of additional treatment support versus routine support is being evaluated in non-overlapping populations for 3HR, 3HP and 1HP, respectively.
- The closed test approach whereby the effect of additional treatment support will only be formally tested for 3HP if there is evidence that 3HP improves adherence compared to 3HR with routine treatment support protects the type I error. This approach will also be used for the assessment of additional treatment support for 1HP.

For peer review only

396 For participants who have collected all prescriptions but are lost to follow-up before completing treatment, the adherence data until the end of allocated period can still be downloaded remotely from the Wisepill monitor box to ascertain whether adequate treatment adherence is achieved; this data will be included in the primary analyses. In sensitivity analyses, the primary outcome will be imputed for these patients using multiple imputation by chained equations (MICE), with imputation to be conducted separately by study arm. Sensitivity analyses will also be performed assuming no drug intake from the last follow-up visit attended.

403 Supplementary analyses will consider different definitions of adequate treatment by varying the minimum proportion of doses required to have been taken, and different allowable time-frames for making up missed doses. In addition, other analysis populations will be considered, including intention-to-treat and per protocol (including only participants who commenced their original allocated trial intervention). Planned exploratory subgroup analyses, will examine outcomes in pre-defined subgroups.

409

410 **Safety reporting**

411 The definitions of the EU Directive 2001/20/EC Article 2 based on the principles of Good Clinical Practice apply to this trial protocol. These definitions are given in Table 3. All Grade 3 or higher adverse events, whether expected or not, will be recorded in the patient's medical notes. All adverse events will be recorded up to week 20. Serious adverse events should be notified to the CTU within 24 hours of the investigator becoming aware of the event from the time of randomisation to the last assessment of adverse events, i.e. week 20. Adverse events will be graded using the DAIDS toxicity grading scale.(30)

418 Participants may be able to claim compensation for injury caused by their participation in the clinical trial in accordance with the insurance policy held at UCL.

420

421 **Monitoring and oversight**

For peer review only - http://bmjopen.bmj.com/site/about/guidelines.xhtml

The trial will be monitored by the MRC-CTU. An Independent Data Monitoring Committee (IDMC) will be formed. The IDMC will review study conduct and safety data regularly. The IDMC will be asked to advise on whether the accumulated data from the trial, together with results from other relevant trials, justify continuing recruitment of further participants. The IDMC will make recommendations to the Programme Steering Committee (PSC) as to whether the trial should continue in its present form.

The PSC has membership from the Trial Management Group (TMG) plus independent members (approved by NIHR), including the Chair and Patient and Public Involvement (PPI) contributors. The role of the PSC is to provide overall supervision for the trial and provide advice through its independent Chair. The ultimate decision for the continuation of the trial lies with the PSC.

Process evaluation

The process evaluation will follow MRC guidance using an embedded, mixed-methods evaluation approach in order to assess acceptability, fidelity, and mechanisms of effects of the interventions. It will be conducted by the research team, working closely with the Intervention Development Group and clinicians delivering the trial.

Patient sample

Patients in the full trial sample will be administered validated questionnaires assessing the psychological characteristics that we predict will mediate the effects of the interventions. Questionnaires will be administered during scheduled clinic appointments at baseline (0 weeks), interim (2 weeks) and treatment completion (either 4 or 12 weeks depending of regimen). Baseline measure will include Beliefs about Medicines Questionnaire (BMQ-Specific/BMQ-General), Perceived Sensitivity to Medicines Scale (PSM-5), Brief illness perceptions questionnaire (BIPQ), The Satisfaction with Information about Medicines Scale (SIMS), Hospital Anxiety and Depression Scale (HADS). At follow-up, participants will complete the BIPQ and BMQ-Specific, and a measure of self-reported adherence (Medication Adherence Report -5 [MARS-5]) and the Treatment intrusiveness Questionnaire (TIQ). A subset of participants will also be approached for a qualitative assessment of their experiences in the trial. Participants in each intervention arm will be purposively

For peer review only - http://bmjopen.bmj.com/site/about/guidelines.xhtml

sampled based on their treatment adherence (10 participants per arm: 5 high adherence, 5 low adherence; total 60 interviews; adherence in line with the primary outcome). Measures will consist of brief, semi-structured interviews.

Staff sample

Healthcare professionals responsible for administering the interventions will be requested to complete a short checklist form following patient randomisation in order to assess intervention fidelity. This will confirm whether each component of the interventions was delivered per protocol. We will also purposively sample 20 service providers to take part in brief, semi-structured interviews (in person or by phone) in order to obtain feedback on the delivery of the intervention and to identify any issues that might enhance delivery in practice. In addition, we will use these interviews to investigate wider contextual issues impacting on delivery. We will also encourage implementing clinicians to report major issues that might compromise intervention delivery during the trial, rather than waiting for a formal interview on trial completion.

Health economic evaluation

This will estimate if changes to LTBI diagnosis and/or treatment are cost-effective from the perspective of the National Health Service, using a health-economic model to synthesise data obtained within the entire RID-TB programme and evidence from other sources. Participants will be asked to complete monthly EQ-5D questionnaires. We will collect information on the costs participants incur in attending appointments within this trial, to allow potential future analysis from a societal perspective.

PATIENT AND PUBLIC INVOLVEMENT (PPI)

The trial was discussed with the charity TB Alert and two community representatives drawn from a migrant charity and a patient previously treated for LTBI. A charity representative and one former patient read versions of the grant proposal and contributed suggestions on study design. At the

For peer review only - http://bmjopen.bmj.com/site/about/guidelines.xhtml

protocol development stage, the following input was sought from TB Alert: study design, treatment support interventions, Participant Information Sheet and Consent form, patient-facing questionnaires used for behavioural studies.

During the trial, we will engage with 1) The RID-TB PPI Advisory Group (PPAG) consisting of members recruited via social media accounts, TB nurses, TB patient advocates, ex-patient contacts and voluntary/community organisations and (2) The TB Action Group (TAG) network of people personally affected by TB. We will seek input for: recruitment, patient/public engagement tools, provision of translated materials on LTBI and access to recruitment sites.

ETHICS AND DISSEMINATION

Ethics approval

Ethics approval has been obtained from the Health Research Authority (HRA) in the UK (20/LO/1097). Any further substantial amendments will be submitted and approved by the main Research Ethics Committee and HRA.

Consent

Participants will be screened and consented at approved trial sites that are authorised by the MRC-CTU to carry out the RID-TB: Treat trial. We will provide potential participants with a copy of the Participant Information Sheet (supplementary materials 1 and 2). We will obtain written informed consent to enter into the trial and be randomised after explanation of the aims, methods, benefits and potential hazards of the trial before any trial-specific procedures are performed. Additional consent to participate in ancillary studies will be sought.

Dissemination

We will report findings of the trial through publications in national and international conferences as well as in peer-reviewed journals. We will follow publication policies used for clinical trials coordinated by the MRC CTU. All headline authors in any publication arising from the main study or

For peer review only - http://bmjopen.bmj.com/site/about/guidelines.xhtml

substudies must have made a substantive academic or project management contribution to the work that is being presented. Findings will be also disseminated via TB Alert, Treatment Action Group, social media, and institutional websites.

Data availability statement

Trial data will be available for sharing by request after the primary publication upon approval by the Trial Management Group.

Trial status

The trial has not yet started recruitment. We expect to start recruitment on 1 September 2022 and the trial will close when all participants have completed follow-up (i.e. 12 months after initiation of treatment), record linkage to ascertain TB has been finished, and after the trial database is locked, which is anticipated to be within 3 months after information on primary and secondary outcomes have been collected.

Protocol version and date

This protocol is an abbreviated version of the protocol version 3.0, October 2020.

Acknowledgements

We thank the NIHR programme officers, UCL-NIHR Patient and Public Involvement Advisory Group, MRC CTU at UCL Protocol Review Committee, and the independent Programme Steering Committee for their support and inputs during the development of the protocol.

Authors contributions

IA and MXR conceived the study. IA and MXR led the application to secure funding. IA, MXR, TD, YH, HB, JC, MF, ALC, AG, VH, EOP, JS, KS, HLB, AC, CG, RH, MJ, HK, ML, MM, PJW, DZ contributed to developing the study design. CL, TD and AMC provided statistical oversight. MXR,

For peer review only - http://bmjopen.bmj.com/site/about/guidelines.xhtml

TD, and YH drafted and revised the manuscript. All authors contributed critical intellectual input and approved the final manuscript.

Funding

The protocol reported in this publication was supported by the NIHR (RP-PG-0217-20009) and will receive support from the NIHR Clinical Research Network (NIHR CRN). Additional funding for patient and public involvement was provided by the UCLH/UCL Biomedical Research Centre Patient & Public Involvement bursary fund. AMC and TD are supported by MRC Grant: MC_UU_12023/27.

Competing interests

None declared.

References

1. Lönnroth K, Migliori GB, Abubakar I, et al. Towards tuberculosis elimination: an action framework for low-incidence countries. European Respiratory Journal. 2015;45:928-52.

2. World Health Organization. Call to Action 2.0: A global drive to scale up TB. https://cdn.who.int/media/docs/default-source/hq-tuberculosis/call-to-action-2.0-a-global-drive-to-scale-up-tb-prevention.pdf?sfvrsn=6f938b38_7&download=trueT_scaleup.pdf?u=f093a7c38a3780cd9504f8d9d&id=135bdee6af&e=09bced52fa Accessed 8 July 2021.

3. Public Health England. Collaborative tuberculosis strategy for England: 2015 to 2020. 2015.

4. Hirsch-Moverman Y, Shrestha-Kuwahara R, Bethel J, et al. Latent tuberculous infection in the United States and Canada: who completes treatment and why? The international journal of tuberculosis and lung disease : the official journal of the International Union against Tuberculosis and Lung Disease. 2015;19:31-8.

5. Saunders MJ, Koh GC, Small AD, et al. Predictors of contact tracing completion and outcomes in tuberculosis: a 21-year retrospective cohort study. The international journal of

For peer review only - http://bmjopen.bmj.com/site/about/guidelines.xhtml

tuberculosis and lung disease : the official journal of the International Union against Tuberculosis and Lung Disease. 2014;18:640-6.

6. Public Health England: LTBI testing and treatment programme for migrants: 2020 report.

7. Public Health England: Tuberculosis in England. 2020 report.

8. Public Health England: LTBI testing and treatment programme for migrants 2020: data tables.

9. Clifford S, Barber N, Horne R. Understanding different beliefs held by adherers, unintentional nonadherers, and intentional nonadherers: Application of the Necessity–Concerns Framework. Journal of psychosomatic research. 2008;64:41-6.

10. NICE. Medicines Adherence: Involving Patients in Decisions about Prescribed Medicines and Supporting Adherence: Full Guideline. Clinical Guideline CG76. 2009.

11. Horne R, Cooper V, Wileman V, et al. Supporting adherence to medicines for long-term conditions: A perceptions and practicalities approach based on an extended common-sense model. European Psychologist. 2019;24:82-96.

12. Liu X, Lewis JJ, Zhang H, et al. Effectiveness of Electronic Reminders to Improve Medication Adherence in Tuberculosis Patients: A Cluster-Randomised Trial. PLoS Med. 2015;12:e1001876.

13. Holzschuh EL, Province S, Johnson K, et al. Use of Video Directly Observed Therapy for Treatment of Latent Tuberculosis Infection - Johnson County, Kansas, 2015. MMWR Morbidity and mortality weekly report. 2017;66:387-9.

14. Lam CK, McGinnis Pilote K, Haque A, et al. Using Video Technology to Increase Treatment Completion for Patients With Latent Tuberculosis Infection on 3-Month Isoniazid and Rifapentine: An Implementation Study. Journal of medical Internet research. 2018;20:e287.

15. World Health Organization. Overcoming key barriers to scale up tuberculosis preventive treatment (TPT) A Call to Action. https://www.who.int/tb/CalltoactionTPT_scaleup.pdf?u=f093a7c38a3780cd9504f8d9d&id=135bdee6af&e=09bced52fa Accessed 18 May 2021.

16. Sterling TR, Villarino ME, Borisov AS, et al. Three months of rifapentine and isoniazid for latent tuberculosis infection. The New England journal of medicine. 2011;365:2155-66.

For peer review only - http://bmjopen.bmj.com/site/about/guidelines.xhtml

17. Zenner D, Beer N, Harris RJ, et al. Treatment of Latent Tuberculosis Infection: An Updated Network Meta-analysis. Ann Intern Med. 2017;167:248-55.

18. Swindells S, Ramchandani R, Gupta A, et al. One Month of Rifapentine plus Isoniazid to Prevent HIV-Related Tuberculosis. The New England journal of medicine. 2019;380:1001-11.

19. Horne R, Chapman SC, Parham R, et al. Understanding patients' adherence-related beliefs about medicines prescribed for long-term conditions: a meta-analytic review of the Necessity-Concerns Framework. PloS one. 2013;8:e80633.

20. Surey J, Stagg HR, Yates TA, et al. An open label, randomised controlled trial of rifapentine versus rifampicin based short course regimens for the treatment of latent tuberculosis in England: the HALT LTBI pilot study. BMC Infectious Diseases. 2021;21:90.

21. Public Health England: Latent TB Testing and Treatment for Migrants. A practical guide for commissioners and practitioners. 2015.

22. evriMED1000 Dispenser https://www.wisepill.com/wisepill-rm1000 Accessed 9 July 2021 [

23. Martinson NA, Barnes GL, Moulton LH, et al. New Regimens to Prevent Tuberculosis in Adults with HIV Infection. New England Journal of Medicine. 2011;365:11-20.

24. Sterling TR, Villarino ME, Borisov AS, et al. Three Months of Rifapentine and Isoniazid for Latent Tuberculosis Infection. New England Journal of Medicine. 2011;365:2155-66.

25. Office for National Statistics. Families and households in the UK: 2020. https://www.ons.gov.uk/peoplepopulationandcommunity/birthsdeathsandmarriages/families/bulletins/familiesandhouseholds/2020 accessed 15 Sep 2021.

26. Public Health England: Tuberculosis in England. 2019 report.

27. Howard DR, Brown JM, Todd S, et al. Recommendations on multiple testing adjustment in multi-arm trials with a shared control group. Stat Methods Med Res. 2018;27:1513-30.

28. Choodari-Oskooei B, Bratton DJ, Gannon MR, et al. Adding new experimental arms to randomised clinical trials: Impact on error rates. Clin Trials. 2020;17:273-84.

29. Wason JMS, Stecher L, Mander AP. Correcting for multiple-testing in multi-arm trials: is it necessary and is it done? Trials. 2014;15:364.

For peer review only

30. U.S. Department of Health and Human Services, National Institutes of Health, National Institute of Allergy and Infectious Diseases, Division of AIDS. Division of AIDS (DAIDS) Table for Grading the Severity of Adult and Pediatric Adverse Events, Corrected Version 2.1. [July 2017]. Available from: https://rsc.niaid.nih.gov/sites/default/files/daidsgradingcorrectedv21.pdf Accessed 11 September 2020.

For peer review only

For peer review only - http://bmjopen.bmj.com/site/about/guidelines.xhtml

615 **Box 1. Study inclusion and exclusion criteria**

Inclusion criteria

1. Aged ≥16 years to ≤65 at screening
2. LTBI diagnosis defined on the basis of all of the following:
   (a) a positive result on an Interferon Gamma Release Assay (IGRA), Tuberculin Skin Test (TST) or C-Tb skin test and
   (b) negative TB symptoms at screening and
   (c) no signs of active TB on a Chest X-ray
3. Eligible for LTBI treatment at TB clinics and national LTBI screening services based on NICE guidelines, which means having one or more of the following :
   - Recent infection (contact tracing);
   - New entrants at risk (i.e., those that immigrated < 5 years from countries with a high incidence of TB, which is defined as ≥ 40 cases/100,000 population); or
   - Individuals who are assessed in the TB clinic for latent TB testing, or have been referred for treatment following testing by specialities or departments within primary or secondary care settings
4. Agree to LTBI treatment
5. Willing and able to provide written informed consent

Exclusion criteria

1. Patients weighing <30 kg.
2. Need for medications that cannot be safely taken together with study drugs (e.g. protease inhibitors in people living with HIV and people with refractory epilepsy taking phenytoin/carbamazepine)
3. Any medical condition deserving priority of treatment (such as: porphyria, malabsorption syndromes, Clostridium difficile-Associated Diarrhoea and other conditions)
4. History of sensitivity/intolerance to isoniazid or rifamycins
5. Individuals with documented liver disease, defined as:
   - LFT (ALT/AST/bilirubin) over three times upper limit of normal (ULN) at baseline. This reflects normal clinical practice. For participant safety, liver function tests are carried on a regular basis. One abnormal value prevents the patient from participating on the study.
   - Clinical diagnosis of cirrhosis (jaundice, hematemesis, ascites or previous episodes of liver encephalopathy),
   - HbsAg positive or HCV antibody positive and deemed ineligible for LTBI treatment by the clinician
6. Intending to move outside of the treatment locality within 20 weeks of starting treatment
7. Individuals who would usually be offered LTBI treatment under Directly Observed Therapy (DOT) as part of enhanced case management in complex cases such as those from under-served groups (such as people who are homeless, misuse substances, have been in prison or who are vulnerable migrants).
8. Use of another experimental investigational medicinal product that is likely to interfere with the study medication within 3 months of study enrolment.
9. Women who are breastfeeding, pregnant, or of childbearing potential who do not agree to use an effective method of contraception from the time consent is signed until 4 weeks after treatment discontinuation or completion. Males whose partners are of childbearing potential must also agree to use an effective method of contraception.
10. Women of childbearing potential without a negative urine pregnancy test within 7 days prior to being registered for trial treatment.

616

For peer review only - http://bmjopen.bmj.com/site/about/guidelines.xhtml

Table 1. Doses of study treatment

	Body weight		
ARM 1 and 2: rifampicin plus isoniazid once daily for 90 doses (3 months)	< 50 KG	≥ 50 kg	
	3 x Isoniazid/Rifampicin fixed dose combination (150/100)	2 x Isoniazid/Rifampicin fixed dose combination (300/150)	
ARM 3 and 4: rifapentine plus isoniazid once weekly for 12 doses (3 months)	30 to < 32 KG	32 to < 50 kg	≥ 50 kg
	Rifapentine 600 mg + Isoniazid 15 mg/kg	Rifapentine 750 mg + Isoniazid 15 mg/kg	Rifapentine 900 mg + Isoniazid 15 mg/kg (900 mg maximum)
ARM 5 and 6: rifapentine plus isoniazid once daily for 28 doses (one month)	30 to < 35 kg	35 to ≤ 45 kg	≥ 45kg
	Rifapentine 300 mg + 300mg Isoniazid	Rifapentine 450 mg + 300 mg isoniazid	Rifapentine 600 mg + 300 mg isoniazid

For peer review only

622 **Table 2.** Definition of the estimands for the primary analyses

623

**Attribute**	**Definition**
Treatments	The primary analyses are based on the following comparisons:<br>a) Arm 3 vs Arm 1- ie 3HP + routine treatment support vs 3HR + routine treatment support<br>b) Arm 5 vs Arm 1- ie 1HP + routine treatment support vs 3HR + routine treatment support<br>c) Arm 2 vs Arm 1- ie 3HR + additional treatment support vs 3HR + routine treatment support<br>If comparison (a) shows 3HP improves adherence compared to 3HR, then additional treatment support will be formally tested for 3HP by comparing Arm 4 vs Arm 3 – ie 3HP + additional treatment support vs 3HP + routine treatment support. Additional treatment support will be similarly assessed for 1HP.
Population	Adults aged 16 to 65 years diagnosed with LTBI and eligible for LTBI treatment.
Endpoint	Adequate treatment adherence, defined as taking ≥90% of allocated doses within the allowable time-frame**.**
Intercurrent events	The main intercurrent events and how they will be handled in the estimand are as follows:<br>• Failure to collect all prescriptions- composite and treatment policy strategies lead to same estimated effect.<br>• Early treatment discontinuation for any reason including adverse event(s) and active TB: a treatment policy strategy will be used, ie the participant is considered to have stopped treatment regardless of the occurrence of the intercurrent event.
Population-level summary measure	Risk ratio for adequate treatment adherence comparing the relevant arms.

624

625

626

For peer review only - http://bmjopen.bmj.com/site/about/guidelines.xhtml

For peer review only

627 **Table 3. Definitions of adverse events and reactions**

Term	Definition
Adverse Event (AE)	Any untoward medical occurrence in a patient or clinical trial participant to whom a medicinal product has been administered including occurrences that are not necessarily caused by or related to that product.
Adverse Reaction (AR)	Any untoward and unintended response to an investigational medicinal product related to any dose administered.
Unexpected Adverse Reaction (UAR)	An adverse reaction, the nature or severity of which is not consistent with the information about the medicinal product in question set out in approved Reference Safety Information for that product in the trial.
Serious Adverse Event (SAE) or Serious Adverse Reaction (SAR) or Suspected Unexpected Serious Adverse Reaction (SUSAR)	Any adverse event, adverse reaction or unexpected adverse reaction that:<br>▪ Results in death<br>▪ Is life-threatening*<br>▪ Requires hospitalisation or prolongation of existing hospitalisation**<br>▪ Results in persistent or significant disability or incapacity<br>▪ Consists of a congenital anomaly or birth defect<br>▪ Is another important medical condition***

628 *The term life-threatening in the definition of a serious event refers to an event in which the patient is at risk of death at the time of the event; it does not refer to an event that hypothetically might cause death if it were more severe, for example, a silent myocardial infarction.

632 **Hospitalisation is defined as an inpatient admission, regardless of length of stay, even if the hospitalisation is a precautionary measure for continued observation. Hospitalisations for a pre-existing condition, that has not worsened or for an elective procedure do not constitute an SAE.

636 *** Medical judgement should be exercised in deciding whether an AE or AR is serious in other situations. The following should also be considered serious: important AEs or ARs that are not immediately life-threatening or do not result in death or hospitalisation but may jeopardise the subject or may require intervention to prevent one of the other outcomes listed in the definition above; for example, a secondary malignancy, an allergic bronchospasm requiring intensive emergency treatment, seizures or blood dyscrasias that do not result in hospitalisation or development of drug dependency.

For peer review only

643 **Figures**

644 **Figure 1. Trial schema**

645

646

For peer review only - http://bmjopen.bmj.com/site/about/guidelines.xhtml

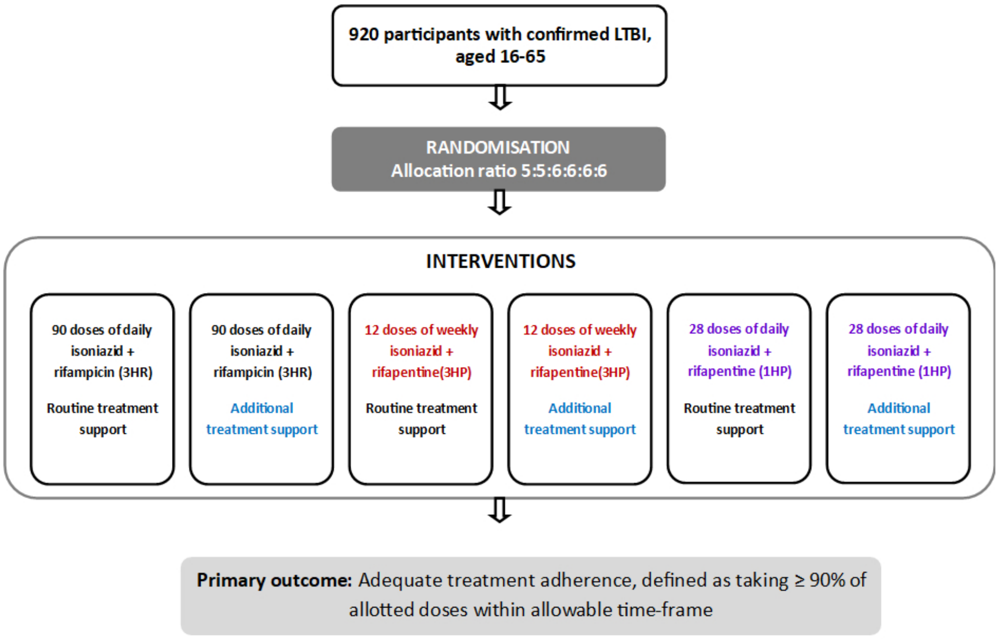

Figure 1.Trial schema

511x332mm (47 x 47 DPI)

Summary Participant Information Sheet for the RID-TB:Treat Clinical Trial

We are inviting you to take part in a research study called RID-TB:Treat.

This study aims to help people complete a course of medicines for treatment of latent tuberculosis infection or LTBI. If you have LTBI, it means you have been infected with the bacteria that cause tuberculosis (TB), but you are not ill and you do not have any symptoms. Treatment for LTBI can prevent TB.

This study is part of RID-TB, a 5–year work programme that includes several linked studies on LTBI. At this stage you are only being asked to take part in RID-TB:Treat.

This page gives you an overview of the study. Please take time to read the whole leaflet carefully so you can decide if you would like to take part. You can share this information sheet and discuss the research study with friends and relatives if you wish.

The study in brief:

This study aims to find out:

- Whether taking different LTBI medicines for a shorter amount of time (one month) than normal (three months), or once a week rather than once a day, makes it easier for people to take all of their medication.
- Whether using a pill box and reminders to take medicines as well as support materials helps people to remember to take their medicine and not miss any doses (we call this adherence support).

In addition, we would also like to find out through the following optional substudies:

- Behavioural substudy: Your thoughts, feelings, and experiences around treatment of LTBI and adherence support
- Health economics substudy: How much treatment of LTBI costs and how the treatment affects your life. This will allow us to see if new LTBI treatment and/or additional adherence support offers value for money for the NHS.

The behavioural and economics substudies are optional, include only people who agree, and will involve completeing simple tick-box questions and interviews for selected individuals.

Key points you need to know:

This study investigates the use of three medicines used in two different combinations. Each combination is given with or without 'adherence support', which includes educational messages and reminders to take medicines.

There are six different groups within the study. You could be allocated to any of these groups:

GROUP 1 - Daily isoniazid + rifampicin for three months (3HR), routine adherence support (standard-of-care)

GROUP 2 - Daily isoniazid + rifampicin for three months (3HR), additional adherence support

GROUP 3 -Weekly isoniazid + rifapentine for three months (3HP), routine adherence support

GROUP 4 - Weekly isoniazid + rifapentine for three months (3HP), additional adherence support

GROUP 5 - Daily isoniazid + rifapentine for one month (1HP), routine adherence support

GROUP 6 - Daily isoniazid + rifapentine for one month (1HP), additional adherence support.

We are studying two new types of LTBI treatment: one month of daily medicines (1HP)

For peer review only - http://bmjopen.bmj.com/site/about/guidelines.xhtml

and three months of medicines which need to be taken once a week (3HP) . These two new types of LTBI treatment will be compared to three months of daily treatment (3HR) usually used in the UK (standard-of-care). We think the medicines should be equally effective and safe. We want to find out whether either of these ways of taking the medicines help you complete a course without missing any doses.

All LTBI medicines, including those used in this trial, can have unwanted side effects. They are usually minor and reversible, if they occur at all. The most common are allergic reactions, flu-like symptoms, headache, skin reactions, diarrhoea, liver problems, nausea, vomiting and a decrease in white blood cell and red blood cell count.

We are also testing whether reminders to take pills and adherence support materials will help you to follow your treatment schedule. You will get a special pill box which will record each time it is opened.

This study will *not* require you to visit the hospital more times than if you were being treated in the usual way for latent TB.

What happens if I am interested in taking part?

If you agree to take part in the study after reading all the information, we will check your medical records. This is to see whether you meet the study entry criteria and check it is safe for you to do take part. We will ask you to sign a consent form and will give you copies of both this information sheet and the consent form. We will also write to your GP to let them know that you have agreed to take part in this research, this is optional and you can opt for your GP not to be informed.

If you do not wish to take part in the study, or if you do not meet the study entry criteria, you are likely to receive the standard-of-care treatment, which is daily antibiotics for three months and usual support to help you remember to take your medicines.

You are free to decide whether to take part in this research study or not. If you choose not to take part, this will not affect the care you receive.

If you do agree, you can stop taking part in the study at any time, without giving a reason.

Please ask your doctor or nurse if there is anything that unclear or if you would like more information.

If you have any questions about this study, please talk to your doctor or nurse:

Name of doctor or nurse:

Hospital Department:

Hospital:

Address:

Tel: 01234 XXX XXX

Email: (if applicable)

1. Why are we doing this study?

This study aims to help people complete prescribed medicines for treatment of latent TB infection (LTBI) and ensure its treatment for LTBI for latent TB works best when taken as prescribed. This study aims to find the best way to support people to take LTBI treatment

What are we trying to find out?

This study aims to find out whether taking different LTBI medicines for a shorter amount of time than normal, or once a week rather than daily, makes it easier for people to take all of their medication and not miss any doses.

We also want to know whether a support package that we have developed which includes a video animation, a pill box and text

For peer review only - http://bmjopen.bmj.com/site/about/guidelines.xhtml

message reminders can help people to take their medicines.

2. What is latent TB?

If you have latent TB infection (LTBI) it means you have been infected with the bacteria that causes tuberculosis (TB), but you are not ill and you do not have any symptoms. If you then become ill with "active" TB disease you could pass TB on to other people. TB bacteria are spread through the air, mainly by coughing. If you want to know more about latent TB talk to the doctor or nurse who is treating you.

How is latent TB usually treated?

Active TB can be cured with a combination of different antibiotics, which need to be taken for many months (at least 6 months). LTBI can be diagnosed and treated to help prevent TB disease from developing. The treatment for latent TB in England is usually 3 months long and fewer drugs are given compared with active disease.

3. Why am I being asked to take part?

You are being asked to take part in the RID-TB:Treat study because you have latent TB and treatment is recommended

Your doctor will perform an assessment and tests that are routinely required before treatment of LTBI for your safety. We will check if you can take partn the study using these results.

To take part in RID-TB:Treat :

- You will be diagnosed with LTBI
- You will be between 16 and 65 years of age
- You will not have signs of active TB (this includes
- You will not have a known allergy to the medicines in the study
- You will not have any liver problems that might mean you can't take the medicines safely (a blood test will be done to check this)
- You will not be pregnant or breastfeeding, or plan on becoming pregnant during the study
- Females who are able to become pregnant (of child-bearing potential) will agree to using contraception whilst on the medications (specifically, an implant or male partners using condoms. Oral contraceptives may be less effective whilst on treatment)
- Male whose female partners are able to become pregnant will agree to using contraception whilst on the medications.

4. What do I need to know about the medicines in this study?

The new LTBI medicine we want to find out more about in this study is called rifapentine, which is given in combination with another medicine called isoniazid. You will only receive rifapentine if you are in Group 3, 4, 5 or 6. There are different ways of taking this tablet: once a day for a month (1HP), or once a week for three months (3HP). It is the same dose each intake. You swallow these tablets within one hour after eating food.

An often-used treatment for LTBI is a medicine combining both rifampicin and isoniazid in a single tablet. You will receive this medicine if you are in Group 1 or 2. This medication is taken once a day, for three months (3HR). You swallow this tablet on an empty stomach (at least 30 minutes before food or 2 hours after food.)

We will also investigate whether additional adherence support helps people to take their medicine. This includes a reminder via SMS message or sound alarm using the pill box and adherence support materials such as video

For peer review only - http://bmjopen.bmj.com/site/about/guidelines.xhtml

animation. This support will be given in addition to usual support by clinicians.

What are the possible side-effects?

All medicines can have unwanted side-effects, including those normally used for LTBI treatment outside of this study. The most common side-effects of rifapentine, rifampicin and isoniazid are: allergic reactions and flu-like symptoms, headache, skin reactions, diarrhoea, liver problems, nausea, vomiting, and decrease in white blood cell and red blood cell count.

A common side effect rifapentine and rifampicin may cause a temporary red-orange staining of body tissues or fluids. This would include skin, teeth, tongue, urine, faeces, saliva, sputum, tears, sweat, and breast milk. Contact lenses or dentures may become permanently stained.

If you become concerned about any side-effects, please tell your doctor or nurse as soon as possible.

5. What are the possible benefits of taking part in this study?

We hope that you will directly benefit from the medicines used in this study and from the tools used to help you with treatment adherence, but we cannot guarantee this. However, the information we will collect from this study will help us to improve future treatments for people like you diagnosed with LTBI in the future.

6. What will I need to do if I take part? Can I definitely take part?

Not everyone may be able to take part in this study. We will first check whether you are suitable for the trial by taking a medical history, checking your symptoms and assessing your physical health. We will also check results of tests which are routinely performed before treatment of latent TB.

If you agree to be part of the trial, you will also be agreeing to:

- Having some tests to check you can take part in the trial. These include, a blood test and a pregnancy test, if you are a woman who is able to become pregnant.
- Receiving any of the six groups of LTBI treatments. To make this a fair test, you cannot choose and must be happy to accept any of the groups of treatment.
- For us to collect your information whilst you are on the study

What if the tests show I can take part?

If these tests show you can take part and you agree to join the RID-TB-Treat study, we will ask you to sign a consent form. There will be six different groups in this study.

Which group will I be in?

It is important that the groups receiving each treatment are as similar as possible at the start of the study. To ensure that this happens, a process called randomisation is used to allocate people to each group. This means, a computer will randomly select which group you are in, like "the toss of a coin". Your doctor will offer you the treatment and adherence support according to your allocated group. Neither you nor your doctor can decide which group you join. You must be willing to accept whichever treatment group you are allocated to.

For peer review only - http://bmjopen.bmj.com/site/about/guidelines.xhtml

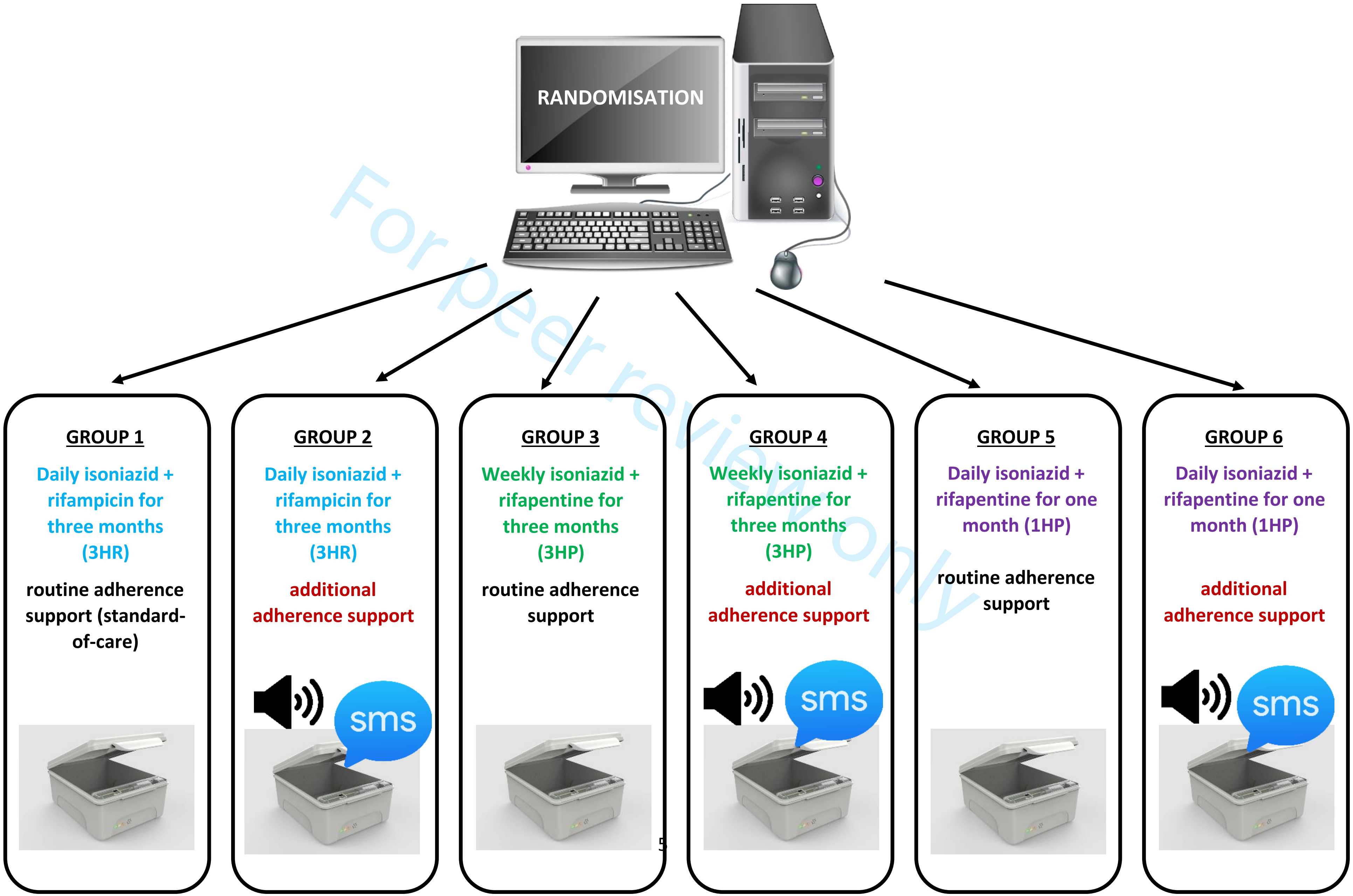

For peer review only

What will happen to me during the study?

Before randomisation, your doctor will again check for signs of active TB and perform a urine pregnancy test. As usual practice, your doctor will provide you with information about latent TB and why it is necessary to take pills as prescribed. As part of the study, you will be given an electronic pill box that automatically records each opening of the box and sends data to the research team via the internet.

If you are allocated to a group with additional adherence support (Groups 2, 4 and 6) you will be asked to set a reminder using the pill box. This can send an SMS message to your phone or sound an alarm at scheduled times or when the box was not opened in a day. You will also be given adherence support materials such as a video animation to watch. Your study team will collect your phone number and will share with selected members of the UCL study team in order to send an SMS message. The study team at UCL will organise the reminders to be sent.

The electronic pill box will collect data on when you open the box and this will be accessed by the team at UCL.

Once you start treatment, you will be required to visit the clinic at week 2 for blood tests to check side effects in accordance with usual care. Additionally, you will have a consultation every month until completion of treatment to assess your health, including checks for signs of active TB and side effects. You will be requested to bring the pill box to check remaining tablets. Blood tests may be performed if your doctor finds it necessary to check liver problems or other side effects. Pregnancy tests will be done at every visit for women of who are able to become pregnant. After completion of treatment, you will receive a phone call every 4 weeks until 20 weeks after start of treatment to check for signs of active TB and side effects.

If you agree to take part in the optional /or substudies you will be required to complete additional questionnaires at every visit.

8. What are the possible disadvantages and risks of taking part?

As with the standard treatment for LTBI, there is a risk of side effects such as liver problems, allergic reactions and flu-like symptoms. The drugs in this study should not be used during pregnancy, therefore women and their partners must use contraception. For women who are able to become pregnant, pregnancy tests will be repeated throughout the study and treatment will be stopped immediately if a participant becomes pregnant.

9. More information about taking part

Do I have to take part in the RID-TB-Treat study?

No, it is up to you to decide whether to take part or not. ,

If you decide not to take part in this study you are likely to receive the standard treatment for LTBI which is antibiotics for three months (daily) and usual care to check and support your adherence. A decision not to take part at any time will not affect the standard of care you receive.

Will I get back any travel costs?

There will not be any reimbursement for travel costs because this study will *not* require you to visit the hospital more times than if you were being treated in the usual way for latent TB.

For peer review only - http://bmjopen.bmj.com/site/about/guidelines.xhtml

Can I stop taking part after I've joined the study?

You can stop taking part in all of this study, or any part of it, at any time and without giving a reason. However, you must talk to your study doctor or nurse first. They can advise you about any concerns you may have.

If you decide to stop taking your study treatment, we will need to continue collecting information about you. This is important because it helps us to ensure that the results of the study are reliable.

If you stop taking part in this study you are likely to receive the standard treatment. A decision to stop taking part at any time will not affect the standard of care you receive.

How will my personal information be used?

University College London (UCL) is the sponsor for this study, based in the United Kingdom. University College London (UCL) will be using information from you and your medical records in order to undertake this study and will act as data controller for this study. University College London (UCL) will be responsible for looking after your information and using it properly, and will keep identifiable information about you for 25 years after the study has finished.

Your rights to access, change or move your information are limited, as we need to manage your information in specific ways in order for the research to be reliable and accurate. If you withdraw from the study, we will keep the information about you that we have already obtained. To safeguard your rights, we will use the minimum personally identifiable information possible.

You can find out more about how we use your information at **www.ctu.mrc.ac.uk/general/privacy-policy**

How will my data be stored and collected?

Your hospital will collect information from you and your medical records for this research study in accordance with our instructions. Your hospital will use your name, NHS number and contact details to: contact you about the research study, make sure that relevant information about the study is recorded for your care, and to oversee the quality of the study.

UCL will collect information about you for this research study from your hospital, NHS Digital and Public Health England (PHE). This information will include your name, postcode and NHS number and health information. Health information is regarded as a special category of information as defined by the General Data Protection Regulation (GDPR). We will use this information to check whether you develop active TB or become pregnant up until one year after study treatment (https://digital.nhs.uk/).

Where information could identify you, the information will be held securely with strict arrangements about who can access the information.

Future research

When you agree to take part in a research study, the information about your health and care may be provided to researchers running other research studies in this and other organisations. They may be universities, NHS institutions or companies involved in health and care research in this country or abroad. Your information will only be used by organisations and researchers to conduct research in accordance with relevant legislation, ethics and NHS research policy requirements.

We won't share information that can identify you with others. The information will only be used for the purpose of health and care research, and cannot be used to contact you or

For peer review only
For peer review only - http://bmjopen.bmj.com/site/about/guidelines.xhtml

to affect your care. It will not be used to make decisions about future services available to you, such as insurance. If there is a risk that you can be identified, your data will only be used in research that has been independently reviewed by an ethics committee.

What will happen to the results of the RID-TB:Treat study?

When the study is completed, we will publish a summary of the results on the website of the MRC CTU at UCL: http://www.ctu.mrc.ac.uk/

We will also publish the results in a medical journal, so that other doctors can see them. You can ask your doctor for a copy of any publication. Your identity and any personal details will be kept confidential. No named information about you will be published in any report of this study.

Who is organising and funding the study?

This study is organised by the MRC CTU at UCL on behalf of The Whittington NHS Trust. The MRC CTU at UCL has run trials for many years. The study coordination, data collection and analysis and administration will be provided by the MRC CTU at UCL. You can find out more about us at www.ctu.mrc.ac.uk

Your doctor is not receiving any money or other payment for asking you to be part of the study. UCL has overall responsibility for the conduct of the study. We are responsible for ensuring the study is carried out ethically and in the best interests of the study participants. A patient representative has been involved in the design of this study and in writing of this information.

Who has reviewed the RID-TB-Treat study?

The study has been reviewed by scientists. It has been approved by the Research Ethics Committee of London Riverside and the National Institute of Health Research (NIHR) who are the funders of the study. It has been authorised by the Medicines and Healthcare products Regulatory Agency (MHRA), as well as by the NHS Health Research Authority (HRA) and the hospital's Research and Development Office.

What if new information becomes available during the course of the study?

Sometimes during a study, new information becomes available about the medicines and procedures that are being studied. If this happens your doctor will tell you about it and discuss with you whether you want to continue the study. If you decide to stop taking part, your doctor will arrange for your care to continue outside of the study.

Your doctor might also suggest that it is in your best interest to stop taking part in the study. They will explain the reasons and arrange for your care to continue outside the study.

What happens if the RID-TB-Treat study stops early?

Very occasionally a study is stopped early. If it happens, the reasons will be explained to you and your doctor will arrange for your care to continue outside of the study.

What if something goes wrong for me?

If you have any concerns about the way you have been approached or treated during the study, please talk to your study doctor or nurse. If you are still unhappy, or if you wish to complain, please use the normal NHS complaints process.

If you are harmed by taking part in the study, or if you are harmed because of someone's negligence, then you may be able to take legal action. The study is covered by the sponsor's

For peer review only - http://bmjopen.bmj.com/site/about/guidelines.xhtml

For peer review only

insurance. Further information can be obtained from the study team on request.

10. Contacts for further information

If you want further information about the RIDTB-Treat study, contact your study doctor or nurse (see below).

[Insert address and telephone number of study doctor and/or nurse]

Thank you for taking the time to consider taking part in this study.

RID-TB:Treat protocol version 3.0, 23-Oct-2020

For peer review only - http://bmjopen.bmj.com/site/about/guidelines.xhtml

(To be presented on local headed paper)

RID-TB:Treat Informed Consent Form
Version 1.0, 26-Aug-2020
IRAS ID: 282304

Centre Name & Number	
Patient ID Number	
Name of Researcher	

#		Initial to Agree	
1	I have read and understood the information sheet for the RID-TB:Treat research study **[Version 1.0, 26-Aug-2020]** and have been given a copy to keep. I have had the chance to ask questions about the project and discuss it with the study staff. I have received answers to all of my questions.		
2	I understand that my medical notes may be looked at by individuals from the Medical Research Council (MRC) Clinical Trials Unit (CTU), or from regulatory authorities where it is relevant to my taking part in this research. I give permission for these individuals to access my records. I understand that my confidentiality will be maintained.		
3	I understand that participation in this trial is voluntary and that I am free to withdraw from the trial at any time, without giving any reason and without my medical care or legal rights being affected.		
4	I understand that I may not benefit directly by participating in this study but that the research may help people with this condition in the future.		
5	In order to follow-up on my health status after my participation in the trial, I give permission for my personal details (such as NHS number, name and date of birth) to be used to obtain information about my health status from records held by NHS Digital, Public Health England, the National TB register, or any applicable national or NHS information system. I understand that this information may be obtained about me during the study and after (up to 25 years).		
6	**I agree to take part in the RID-TB:Treat study.**		
**Optional Items:**	***If you wish to give permission, put your initials in the 'Yes' box. If you do not wish to give permission, put your initials in the 'No' box. If you do not agree to any of the following items, you can still take part in the main study.***	**Yes**	**No**
7	I agree for my GP to be informed of my participation in the research study.		
8	I agree to participate in the Behavioural Sub-study and to complete the questionnaires.		
9	I agree to participate in the Health Economics Sub-study and to complete the questionnaires.		

Signatures

Name of Participant (BLOCK CAPITALS)	Date (Day/month/year)	Signature (or thumbprint)
Name of Witness (BLOCK CAPITALS)	Date (Day/month/year)	Signature (if thumbprint used above)
Name of person taking consent (BLOCK CAPITALS)	Date (Day/month/year)	Signature

IMPORTANT: Signed original to be kept in the Investigator Site File
One copy to be given to the participant
One copy to be kept with the participant's medical notes

For peer review only

SPIRIT
STANDARD PROTOCOL ITEMS: RECOMMENDATIONS FOR INTERVENTIONAL TRIALS

SPIRIT 2013 Checklist: Recommended items to address in a clinical trial protocol and related documents*

Section/item	Item No	Description	Addressed on page number
**Administrative information**			
Title	1	Descriptive title identifying the study design, population, interventions, and, if applicable, trial acronym	1
Trial registration	2a	Trial identifier and registry name. If not yet registered, name of intended registry	3
	2b	All items from the World Health Organization Trial Registration Data Set	1-20
Protocol version	3	Date and version identifier	20
Funding	4	Sources and types of financial, material, and other support	21
Roles and responsibilities	5a	Names, affiliations, and roles of protocol contributors	1, 21
	5b	Name and contact information for the trial sponsor	1
	5c	Role of study sponsor and funders, if any, in study design; collection, management, analysis, and interpretation of data; writing of the report; and the decision to submit the report for publication, including whether they will have ultimate authority over any of these activities	21
	5d	Composition, roles, and responsibilities of the coordinating centre, steering committee, endpoint adjudication committee, data management team, and other individuals or groups overseeing the trial, if applicable (see Item 21a for data monitoring committee)	18

For peer review only

**Introduction**			
Background and rationale	6a	Description of research question and justification for undertaking the trial, including summary of relevant studies (published and unpublished) examining benefits and harms for each intervention	6-8
	6b	Explanation for choice of comparators	6-8
Objectives	7	Specific objectives or hypotheses	8-9
Trial design	8	Description of trial design including type of trial (eg, parallel group, crossover, factorial, single group), allocation ratio, and framework (eg, superiority, equivalence, noninferiority, exploratory)	9-10
**Methods: Participants, interventions, and outcomes**			
Study setting	9	Description of study settings (eg, community clinic, academic hospital) and list of countries where data will be collected. Reference to where list of study sites can be obtained	10
Eligibility criteria	10	Inclusion and exclusion criteria for participants. If applicable, eligibility criteria for study centres and individuals who will perform the interventions (eg, surgeons, psychotherapists)	10
Interventions	11a	Interventions for each group with sufficient detail to allow replication, including how and when they will be administered	10-12
	11b	Criteria for discontinuing or modifying allocated interventions for a given trial participant (eg, drug dose change in response to harms, participant request, or improving/worsening disease)	13-14
	11c	Strategies to improve adherence to intervention protocols, and any procedures for monitoring adherence (eg, drug tablet return, laboratory tests)	11-12
	11d	Relevant concomitant care and interventions that are permitted or prohibited during the trial	11
Outcomes	12	Primary, secondary, and other outcomes, including the specific measurement variable (eg, systolic blood pressure), analysis metric (eg, change from baseline, final value, time to event), method of aggregation (eg, median, proportion), and time point for each outcome. Explanation of the clinical relevance of chosen efficacy and harm outcomes is strongly recommended	13-14
Participant timeline	13	Time schedule of enrolment, interventions (including any run-ins and washouts), assessments, and visits for participants. A schematic diagram is highly recommended (see Figure)	12-13, Figure 1

For peer review only - http://bmjopen.bmj.com/site/about/guidelines.xhtml

Sample size	14	Estimated number of participants needed to achieve study objectives and how it was determined, including clinical and statistical assumptions supporting any sample size calculations	14
Recruitment	15	Strategies for achieving adequate participant enrolment to reach target sample size	10

Methods: Assignment of interventions (for controlled trials)

Allocation:

Sequence generation	16a	Method of generating the allocation sequence (eg, computer-generated random numbers), and list of any factors for stratification. To reduce predictability of a random sequence, details of any planned restriction (eg, blocking) should be provided in a separate document that is unavailable to those who enrol participants or assign interventions	15
Allocation concealment mechanism	16b	Mechanism of implementing the allocation sequence (eg, central telephone; sequentially numbered, opaque, sealed envelopes), describing any steps to conceal the sequence until interventions are assigned	15
Implementation	16c	Who will generate the allocation sequence, who will enrol participants, and who will assign participants to interventions	15
Blinding (masking)	17a	Who will be blinded after assignment to interventions (eg, trial participants, care providers, outcome assessors, data analysts), and how	NA
	17b	If blinded, circumstances under which unblinding is permissible, and procedure for revealing a participant's allocated intervention during the trial	NA

Methods: Data collection, management, and analysis

Data collection methods	18a	Plans for assessment and collection of outcome, baseline, and other trial data, including any related processes to promote data quality (eg, duplicate measurements, training of assessors) and a description of study instruments (eg, questionnaires, laboratory tests) along with their reliability and validity, if known. Reference to where data collection forms can be found, if not in the protocol	15-16
	18b	Plans to promote participant retention and complete follow-up, including list of any outcome data to be collected for participants who discontinue or deviate from intervention protocols	15-16

For peer review only - http://bmjopen.bmj.com/site/about/guidelines.xhtml

Data management	19	Plans for data entry, coding, security, and storage, including any related processes to promote data quality (eg, double data entry; range checks for data values). Reference to where details of data management procedures can be found, if not in the protocol	15-16
Statistical methods	20a	Statistical methods for analysing primary and secondary outcomes. Reference to where other details of the statistical analysis plan can be found, if not in the protocol	16-17
	20b	Methods for any additional analyses (eg, subgroup and adjusted analyses)	16-17
	20c	Definition of analysis population relating to protocol non-adherence (eg, as randomised analysis), and any statistical methods to handle missing data (eg, multiple imputation)	16-17
**Methods: Monitoring**			
Data monitoring	21a	Composition of data monitoring committee (DMC); summary of its role and reporting structure; statement of whether it is independent from the sponsor and competing interests; and reference to where further details about its charter can be found, if not in the protocol. Alternatively, an explanation of why a DMC is not needed	18
	21b	Description of any interim analyses and stopping guidelines, including who will have access to these interim results and make the final decision to terminate the trial	18
Harms	22	Plans for collecting, assessing, reporting, and managing solicited and spontaneously reported adverse events and other unintended effects of trial interventions or trial conduct	17
Auditing	23	Frequency and procedures for auditing trial conduct, if any, and whether the process will be independent from investigators and the sponsor	18
**Ethics and dissemination**			
Research ethics approval	24	Plans for seeking research ethics committee/institutional review board (REC/IRB) approval	20
Protocol amendments	25	Plans for communicating important protocol modifications (eg, changes to eligibility criteria, outcomes, analyses) to relevant parties (eg, investigators, REC/IRBs, trial participants, trial registries, journals, regulators)	20

For peer review only - http://bmjopen.bmj.com/site/about/guidelines.xhtml

Consent or assent	26a	Who will obtain informed consent or assent from potential trial participants or authorised surrogates, and how (see Item 32)	20
	26b	Additional consent provisions for collection and use of participant data and biological specimens in ancillary studies, if applicable	20
Confidentiality	27	How personal information about potential and enrolled participants will be collected, shared, and maintained in order to protect confidentiality before, during, and after the trial	16
Declaration of interests	28	Financial and other competing interests for principal investigators for the overall trial and each study site	21
Access to data	29	Statement of who will have access to the final trial dataset, and disclosure of contractual agreements that limit such access for investigators	20
Ancillary and post-trial care	30	Provisions, if any, for ancillary and post-trial care, and for compensation to those who suffer harm from trial participation	18l
Dissemination policy	31a	Plans for investigators and sponsor to communicate trial results to participants, healthcare professionals, the public, and other relevant groups (eg, via publication, reporting in results databases, or other data sharing arrangements), including any publication restrictions	20
	31b	Authorship eligibility guidelines and any intended use of professional writers	21
	31c	Plans, if any, for granting public access to the full protocol, participant-level dataset, and statistical code	20
**Appendices**			
Informed consent materials	32	Model consent form and other related documentation given to participants and authorised surrogates	Supplementary
Biological specimens	33	Plans for collection, laboratory evaluation, and storage of biological specimens for genetic or molecular analysis in the current trial and for future use in ancillary studies, if applicable	NA

*It is strongly recommended that this checklist be read in conjunction with the SPIRIT 2013 Explanation & Elaboration for important clarification on the items. Amendments to the protocol should be tracked and dated. The SPIRIT checklist is copyrighted by the SPIRIT Group under the Creative Commons "Attribution-NonCommercial-NoDerivs 3.0 Unported" license.

For peer review only - http://bmjopen.bmj.com/site/about/guidelines.xhtml
